# The formation of tonalitic and granodioritic melt from Venusian basalt

**DOI:** 10.1038/s41598-022-05745-3

**Published:** 2022-01-31

**Authors:** Yao Jui Wang, J. Gregory Shellnutt, Jennifer Kung, Yoshiyuki Iizuka, Yu-Ming Lai

**Affiliations:** 1 Department of Earth Science, National Cheng Kung University, No. 1 University Road, Tainan, 701 Taiwan; 2 Department of Earth Sciences, National Taiwan Normal University, 88 Tingzhou Road Section 4, Taipei, 11677 Taiwan; 3 Institute of Earth Sciences, Academia Sinica, 128 Academia Road Section 2, Taipei, 11529 Taiwan

**Keywords:** Planetary science, Solid Earth sciences

## Abstract

The crust of Venus is composed of the low lying volcanic planitiae and the elevated, deformed tesserae. It is thought that the tesserae may be composed of silicic igneous rocks and that it may resemble proto-continental crust. The initial development of terrestrial continental crust is likely due to melting and deformation of primitive mafic crust via mantle-plume upwelling and collisional plate processes. Unlike Earth, the lithosphere of Venus is not divided into plates and therefore evolved continental crust, if present, developed primarily by melting of pre-existing mafic crust. Here, we report the results of high pressure equilibrium partial melting experiments using a parental composition similar to the basalt measured at the Venera 14 landing site in order to determine if silicic melts can be generated. It was found that at pressures of 1.5 GPa and 2.0 GPa and temperatures of 1080 °C, 1090 °C, and 1285 °C that tonalitic and granodioritic melts can be generated. The experimental results indicate that silicic rocks may be able to form in the crust of Venus providing the thermal regime is suitable and that the lower crust is basaltic. The implication is that the older, thicker regions of Venusian crust may be partially composed of silicic igneous rocks.

## Introduction

Venus is similar to Earth in size, composition, and mass however it appears that the crust of both planets evolved along very different pathways^1,2^. The Earth is the only planet in the Solar System known to have differentiated silicic and alumina-rich (sialic) continental crust^3^. The formation and growth of continental crust is not completely understood, but it likely developed as a consequence of melting and deformation of an initially primitive mafic crust via mantle plume-related magmatism and collisional plate processes^3–5^. The oldest crust of the Earth is composed of the low metamorphic grade granite-greenstone belts and the high metamorphic grade granulite-gneiss belts^6^. Furthermore, a significant component of Archean continental crust is composed of the so-called ‘grey gneisses’ of which the sodic granitoids of this group are collectively referred to as the tonalite-trondhjemite-granodiorite (TTG) series^7–9^. Key to the evolution of terrestrial continental crust is the initiation of plate tectonics which likely began during the Neoarchean (2.8 Ga to 2.5 Ga) although alterative periods are proposed^4,10–14^.

The surface morphology of Venus indicates that plate tectonics did not develop as a globe-encircling network of mid-ocean ridges and subduction zones is not present^15^. In spite of the absence of plate tectonic features, Venus appears to have a relatively young surface, hotspot-related magmatism, and structures indicative of horizontal compressive stress^16–19^. Moreover, approximately 8% of the Venusian surface is composed of morphologically distinct, older, deformed, and elevated (> 2 km above mean planetary radius) domains known as tesserae^20^. It is thought that the tesserae may be analogous to proto-continental terrestrial crust, however there is uncertainty in the preponderance of rock types and compositions and both silicic and mafic rocks have been proposed^1,21–24^.

The surface composition of Venus was measured at seven locations between equatorial and tropical latitudes of the volcanic plains. The data indicate that the surface is composed of mafic rocks that resemble terrestrial tholeiitic and alkalic basalt^25^. Of particular interest is the rock measured at the Venera 14 landing site near Navka Planitia as it is similar to terrestrial tholeiitic basalt that is typical of Archean granite-greenstone belts and within-plate settings^26,27^. The identification of a rock similar to terrestrial tholeiitic basalt has potential implications for the genesis of ancient sialic crust on Venus. Although there is debate on the conditions of melting, the formation of TTG magmas is related to partial melting of mafic or metamafic rocks that resemble tholeiitic basalt of granite-greenstone belts^8,28–30^.

In this work, we present the results of high pressure (> 1 GPa) equilibrium partial melting experiments using a synthetic rock that is similar in composition to the basalt identified at the Venera 14 landing site (Table S1). The melt compositions derived from the parental basalt are tonalitic and granodioritic and bear a resemblance to rocks of the Archean TTG series of Earth. Therefore, it is possible that silicic igneous rocks are a component of the thickened regions of Venusian crust.

## Experimental results

**Figure 1:**
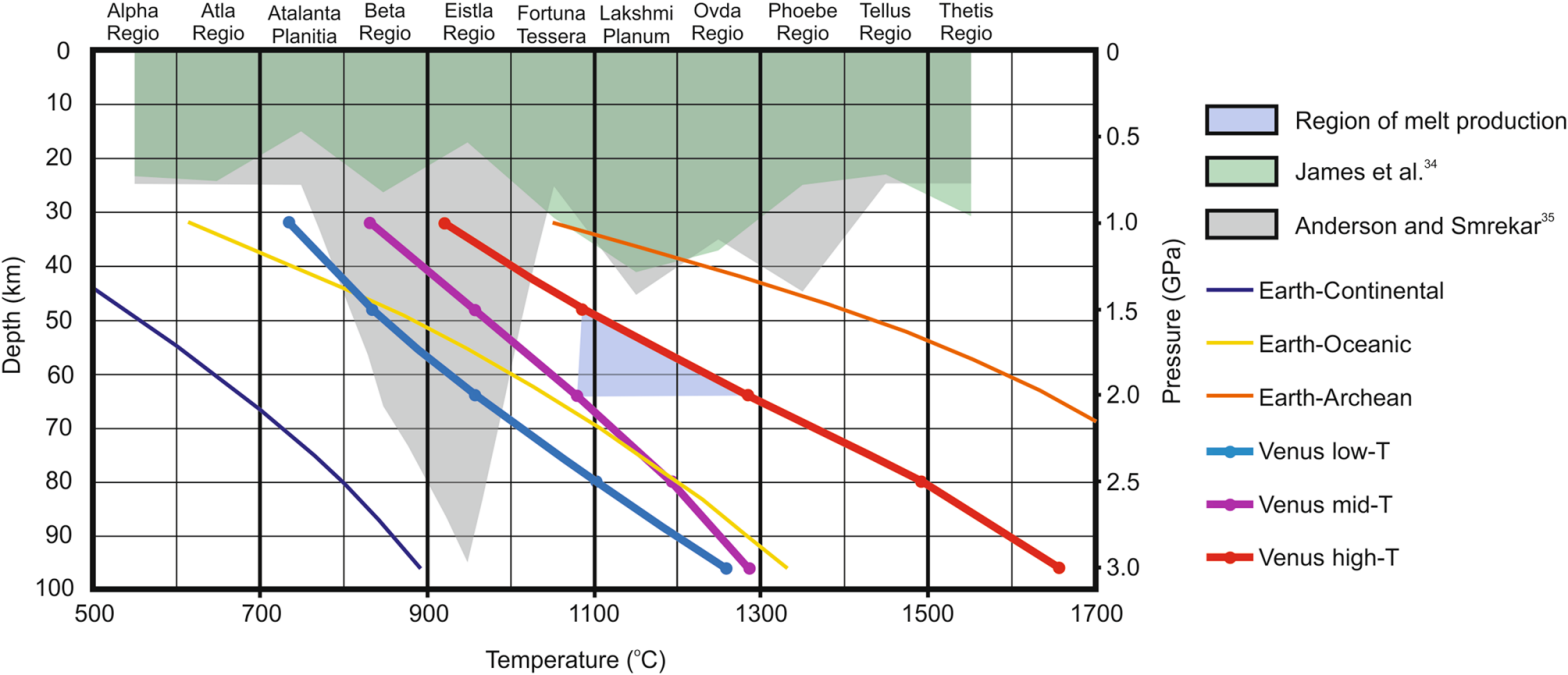
Schematic diagram of Venusian crustal thickness and thermal gradient models. The dark green area is the tesserae crustal thickness estimate by James et al.^34^ and the grey area is the tesserae crustal thickness estimated by Anderson and Smrekar^35^. The geothermal gradients of the Earth’s continental^4^ and oceanic crust^4^ and Archean^4^ conditions are shown in comparison to the estimated single plate thermal gradients of the Venus low-temperature^32^, Venus mid-temperature^31^, and Venus high-temperature^32^ conditions used for this study. The region of melting is show in light blue.

The results of the piston cylinder (1.0 GPa to 1.5 GPa) and large volume press (2.0 GPa to 3.0 GPa) high pressure experiments are summarized in Table S2 of the supplementary material and the methods are described at the end of the paper. The piston cylinder results show that melt, identified as glass, is generated at 1.5 GPa and 1090 °C with a residual mineralogy of orthopyroxene, clinopyroxene, Fe-Ti oxide minerals, plagioclase, and a SiO_2_-phase (Table S3). The lower temperature experiments at 1.5 GPa (960 °C) and 1.0 GPa (920 °C) did not yield a melt. The large volume press results show that melt is generated at 2.0 GPa and 1080 °C and 1285 °C with a residual mineralogy similar to the piston cylinder experiments. The geothermal gradients of the higher (1090 °C and 1285 °C) temperature experiments that produced melt are within the estimated Venus high-temperature (20–23 °C/km) geotherm range whereas the lower temperature experiment was within the estimated Venus mid-temperature (17 °C/km) geotherm range (Fig. 1)^31,32^. All other high pressure experiments at 2.5 GPa and 3.0 GPa failed to produce melt likely because they were performed at the estimated Venus low-temperature and mid-temperature gradient ranges (Fig. 1)^31,32^.

**Figure 2:**
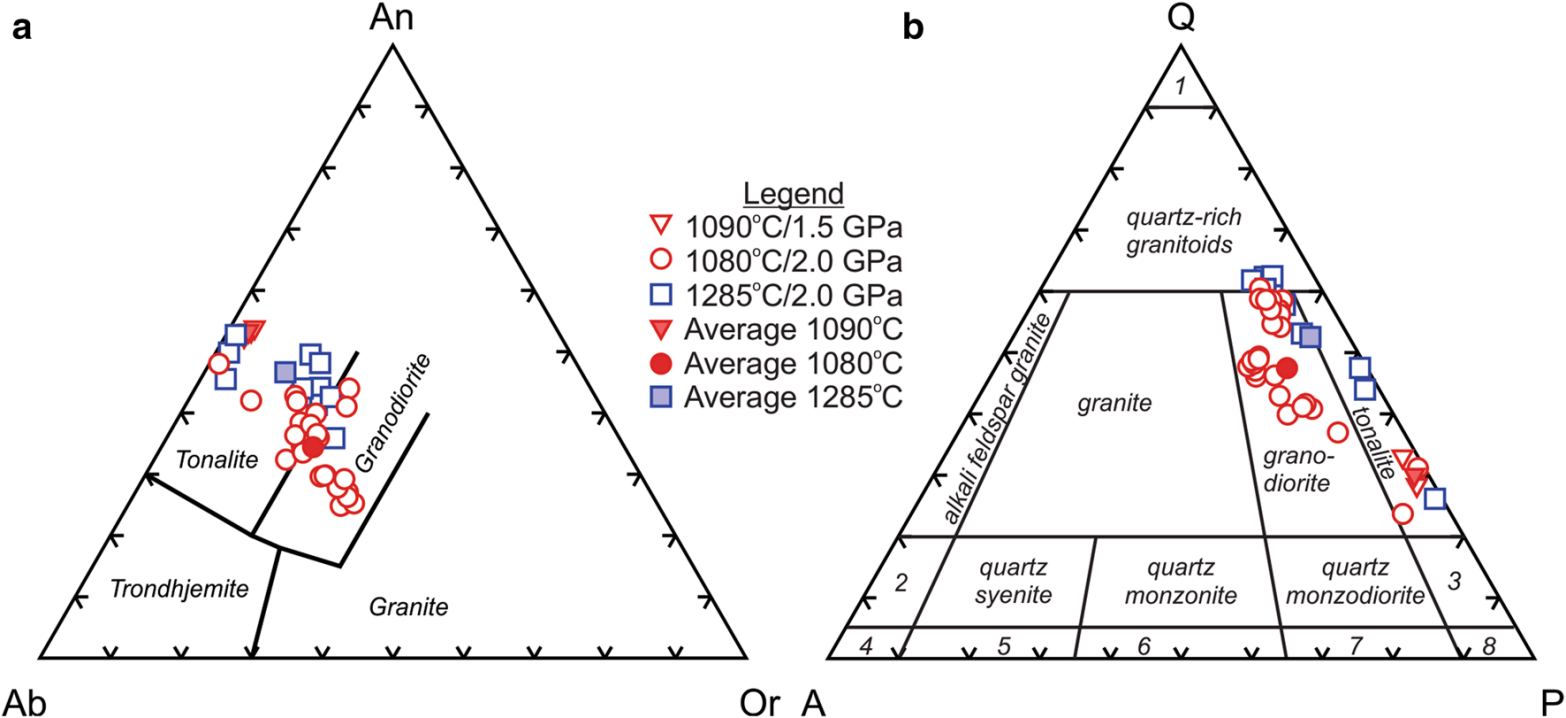
Normative mineralogy classification of the experimental glass compositions. (**a**) CIPW (Cross, Iddings, Pirsson, Washington) normative mineral classification of tonalite-trondhjemite-granodiorite-granite using albite (Ab)-anorthite (An)-orthoclase (Or) proportions. (**b**) Quartz-alkali feldspar-plagioclase (QAP) classification of silicic plutonic rocks using the CIPW normative mineralogy of the experimental glass compositions. 1—quartzolite, 2—quartz alkali feldspar syenite, 3—quartz diorite, 4—alkali feldspar syenite, 5—syenite, 6—monzonite, 7—monzodiorite, 8—diorite. An Fe^3+^/total Fe ratio of 0.3 was used in the normative calculation.

The mineral compositions of the initial rock and the glass compositions and their normative mineralogy are listed in Table S3. The low pressure (1.5 GPa) melt compositions are weakly silicic (SiO_2_ = 64.51 to 66.55 wt%), alumina-rich (Al_2_O_3_ = 17.94 to 19.34 wt%), sodic (K_2_O/Na_2_O = 0.08 to 0.11), and have variable Mg# (11.5 to 40.7). In contrast, the high pressure (2.0 GPa) melt compositions are sodic to weakly potassic (K_2_O/Na_2_O = 0.04 to 1.04), highly silicic (SiO_2_ = 64.99 to 76.52 wt%), but more variable across all elements. The normative albite-anorthite-orthoclase classification of both groups falls within the tonalite-granodiorite range (Fig. 2). Specifically, the lower pressure compositions are exclusively tonalite whereas the high pressure compositions are tonalite and granodiorite.

**Figure 3:**
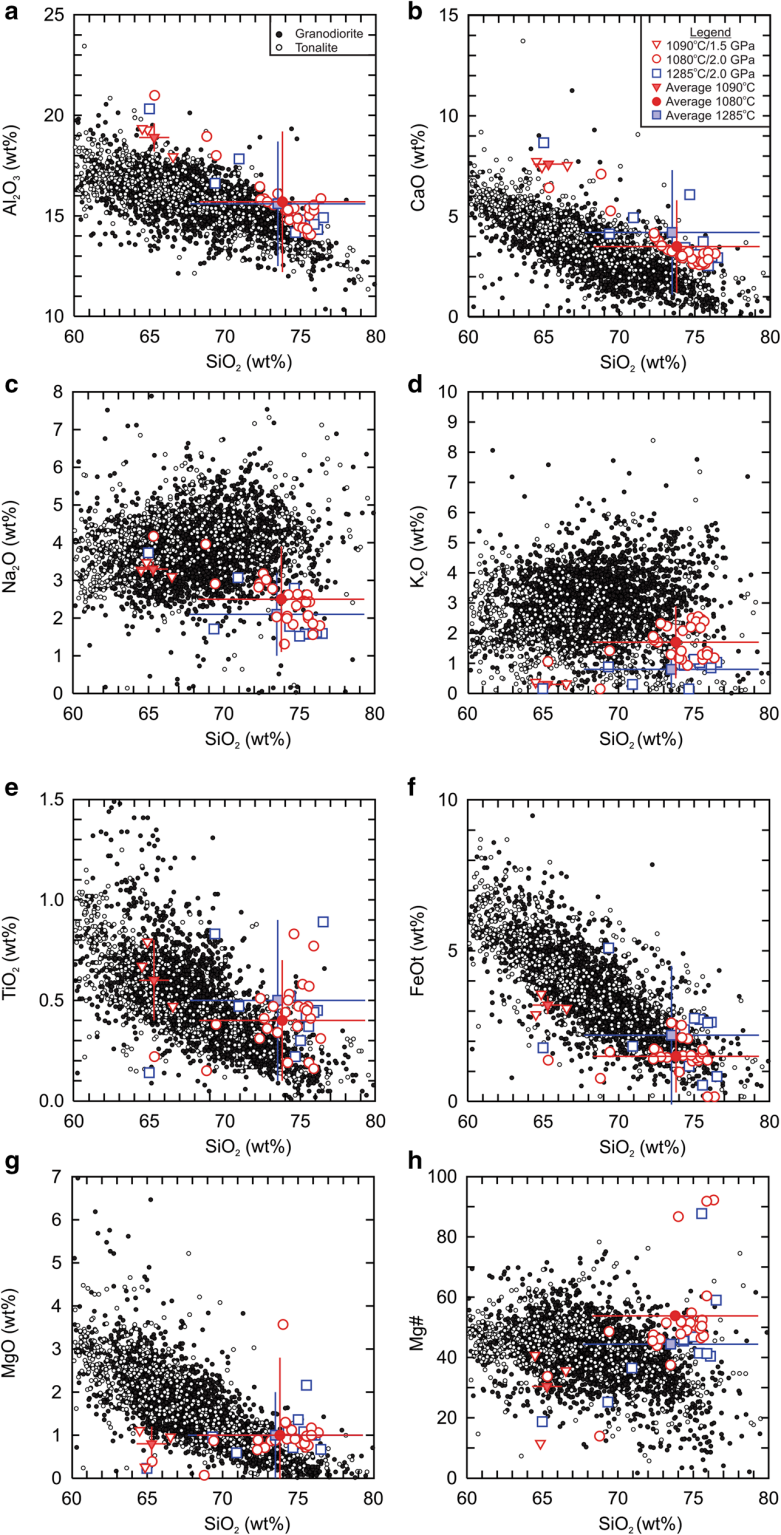
Comparison of the whole rock experimental glass compositions with terrestrial tonalite and granodorite. (**a**) Al_2_O_3_ (wt%) vs. SiO_2_ (wt%), (**b**) CaO (wt%) vs. SiO_2_ (wt%), (**c**) Na_2_O (wt%) vs. SiO_2_ (wt%), (**d**) K_2_O (wt%) vs. SiO_2_ (wt%), (**e**) TiO_2_ (wt%) vs. SiO_2_ (wt%), (**f**) FeOt (wt%) vs. SiO_2_ (wt%), (**g**) MgO (wt%) vs. SiO_2_ (wt%), and (**h**) Mg# ([mol. MgO/(mol. MgO + mol. FeOt)] * 100) vs. SiO_2_ (wt%). The tonalite and granodorite data are compiled from the GEOROC database (http://georoc.mpch-mainz.gwdg.de/georoc/). Only samples with major elemental sum totals of 99 wt% to 101 wt% were selected. All data plotted are recalculated to 100% on an anhydrous basis. FeOt = 0.8998 * Fe_2_O_3_t.

The melt compositions and their average values compare favourably to the range of terrestrial tonalite and granodiorite (Fig. 3). The TiO_2_ and CaO concentrations tend to be a little high but are within the range of terrestrial tonalite and granodiorite, however the concentration of Na_2_O is generally lower and thus the alumina saturation index values are high (mol. Al/Ca + Na + K > 1.25) for some analyses. The overall lower Na_2_O concentrations in the glass could be related to the higher anorthite values (An% = 67.5 to 72.1) in the plagioclase of the initial rock composition. Previous partial melting experiments to understand the formation of TTG rocks with “mafic” starting materials often contain andesine plagioclase (An_50-30_)^28,29^ which is normally associated with intermediate rocks. Alternatively, it is possible that the low Na_2_O concentrations could be an artifact of the anhydrous nature of the experiments or due to the decay of sodium counts during the analytical counting period^29^. Thus, it is possible that the concentration of Na_2_O is, in general, underestimated, but we think the average values of the glass are representative of the melt that is derived from the source rock of the experiments.

## Formation of silicic rocks in the crust of Venus

The experimental results presented in this study show that the melt compositions are similar to terrestrial tonalite and granodiorite and can be generated by partial melting at 1.5 GPa and 2.0 GPa of a rock similar in composition to that encountered at the Venera 14 landing site. Our results are consistent with petrological experiments that use metamafic tholeiitic rocks from greenstone belts and show that the TTG rock series can be derived by partial melting at pressures from < 1.0 to > 2.5 GPa^8,9^. The observed chemical variability within the terrestrial sodic TTG series is mostly due to differences in depth of melting and water concentration as the stabilities of amphibole, rutile, and garnet are important for generating melts that have distinct trace element compositions^8^. Moreover, the source rock mineralogy will influence the melt composition as well. This could be a reason why the melt compositions of the study are a little less sodic as many of the previous experiments used relatively evolved terrestrial basalt (i.e., MgO ≈ 6 wt%; Mg# < 60)^29^ whereas the Venera 14 rock is more primitive (i.e., MgO ≈ 7.5 wt%; Mg# > 60). It is also possible that the anhydrous nature of the experiment played a role in the lower sodium concentrations. Nevertheless, the results of this study have potential implications for the evolution of the Venusian crust.

The tesserae are considered to be the oldest regions of the Venusian crust based on geological relationships and they also appear to be deformed and may have slightly older crater retention ages than the surrounding volcanic plains^16,20,22^. Remote sensing and geochemical mapping surveys suggest that the tesserae could be silicic and/or contain anorthosite^21,22^ and thus, to some extent, may resemble ancient (Archean?) continental crust, but there is uncertainty in the data and alternative explanations are possible. For example, it is proposed that the surface of some tesserae is composed of basalt flows or sedimentary rocks^23,24^. However, this interpretation does not preclude the possibility that the upper to lower middle tesserae crust is more silicic^33^. Crustal thickness estimates (i.e., 25–65 km) of some Venusian tesserae (e.g., Ovda Regio, Fortuna Tesserae, Maxwell Montes, Lakshmi Planum, Beta Regio) are within the range expected for the melting pressure of terrestrial mafic crust that generates silicic igneous rocks at subduction-unrelated settings^8,34–36^. It is entirely possible that the base of the original Venusian crust that generated the tesserae was similar to primitive basalt (i.e., Venera 14) and that it became thicker over time either because of cumulative volcanic eruptions or horizontal lithotectonic stress or a combination of the two^37–40^. If a volcanic plateau or mafic crust achieved a thickness of 25–65 km then the pressure conditions would be sufficient to generate tonalitic and granodioritic magmas at their base providing the thermal regime was suitable. Furthermore, in this environment, the base of a plateau/crust would likely be comparatively dry even though there may have been surface water on Venus in the past^8,29^.

The current interior thermal regime of Venus is unknown but it has been estimated using a number of different methods. Mantle potential temperature (T_P_) estimates using the compositions of the Venera 13, Venera 14, and Vega 2 basalts indicate that Venus either had or may have upper mantle conditions similar to the ambient conditions of modern Earth (i.e., T_P_ = 1350 ± 50 °C) or possibly even close to Archean Earth conditions (i.e., T_P_ ≈ 1600 °C)^41–43^. The estimated eruption temperature of the calculated primitive Venera 14 lava is 1300 ± 60 °C and well within the temperature range (1080 °C to 1285 °C) of our study^43^. The Venusian geothermal gradients in our experiments are based on the estimates by Steinberger et al.^31^ and Armann and Tackely^32^. Perhaps most importantly, the geothermal gradients of the highest temperature melts (1090 °C and 1285 °C) are 20–23 °C/km which is close to the lower bound (> 25 °C/km) of the estimated Archean Earth geotherm but 5–11 °C/km higher than the geothermal gradient of modern terrestrial subduction (12–15 °C/km) zones^29,44^. In other words, the thermal conditions of the highest temperature experiments are similar to those that would be expected from a mantle plume-related intra-plate or anorogenic setting and consistent with the tectonomagmatic regimes that are thought to operate on Venus^18,45,46^. Therefore, we think it is possible that Venusian tonalitic and granodioritic magmas can be generated at the base of thickened mafic crust by a high temperature regime similar to a mantle plume or hotspot.

## Implications for crustal evolution of Venus: analogous to Earth?

Our results supporting the derivation of tonalitic and granodioritic melts by partial melting of Venusian basalt suggest that sialic crust development on Venus may have been similar to pre-plate tectonics Earth^47,48^. The generation of terrestrial sialic crust likely accelerated due to the initiation of modern plate tectonics on Earth as it is intimately associated with the generation of silicic igneous rocks at convergent plate boundaries^5^. Although the initiation of plate tectonics is debated^10,11,13,14^, key to the discussion is the development of the TTG-series as they are mostly restricted to the Archean (> 2.5 Ga) and thought to be generated at both subduction-unrelated and subduction-related tectonic settings^8,49,50^.

It is likely that intra-plate tectonic processes related to mantle upwelling and sinking of dense lithosphere predominated prior to the shift toward subduction-related tectonics sometime during the Archean^11,13,38,39,47^. If this is the case then it would imply that subduction-unrelated TTG rocks likely developed before subduction-related TTG rocks and that their relative proportions were temporally inverse until the end of the Archean when TTG rocks became less common. The secular increase in the Mg# ratio, and Sr, Ni, and Cr concentrations of TTG rocks may reflect temporal changes in the proportion of subduction-unrelated vs. subduction-related TTGs, but this is debated and there are alterative explanations for their secular compositional variation^50–52^.

The Early Archean terrestrial mantle was hotter than the Late Archean mantle and thus it was more likely that upwelling of hot mantle plumes were contributing to proto-continental crust growth with crustal recycling by melting of preexisting mafic crust^4,30,38,48^. Moreover, if mantle upwelling was the primary interior cooling mechanism of the Early Archean Venusian mantle then it would be expected that the same or similar process of crustal growth and recycling by partial melting of preexisting mafic crust would have occurred as well^4,38,40^. Given that the tesserae are thick, older, and distinct lithotectonic domains, it would stand to reason that they may be partially composed of silicic rocks, in their middle to upper crustal regions that were generated by partial melting related to mantle plume magmatism.

## Methods

### Synthetic rock procedure

The starting material was prepared from oxide reagent powders (SiO_2_, TiO_2_, Al_2_O_3_, Fe_2_O_3_, MnO_2_, MgO, CaCO_3_, Na_2_CO_3_, K_2_CO_3_), based on the chemical composition measured at the Venera 14 landing site^25^. The mixture of oxides was heated to the temperature of 1550 °C, and quenched to room temperature to form a homogeneous glass. The chemical compositions of the synthetic Venera 14 basalt and the starting glass composition are tabulated in Table S1. In order to form the initial ‘basaltic rock’ that was used in the partial melting experiments, the starting glass was annealed at 1150 °C for 2 weeks.

### Piston cylinder apparatus

In all experiments, the sample was ground into a fine powder and placed into Au_75_-Pd_25_ capsules. The Au–Pd capsules reduce the escape of iron and control the oxygen fugacity close to the Ni-NiO buffer. The high pressure (1.0 and 1.5 GPa) piston cylinder experiments were carried out using a Quickpress 3.0 piston cylinder apparatus (Depths of the Earth Company). Pressure is generated by pumping a hydraulic ram to force a piston into a pressure plate that consists of concentric layers of hardened-steel around a tungsten carbide core. The area ratio between the ram and the piston is 100:1. The operating machine generates a pressure range between 0.5 and 2.5 GPa (~ 75 km depth) and a maximum temperature of 2200 °C. The pressure-cell apparatus was set to a pressure ten percent higher than the expected pressure. Then, the experiment temperature was raised by the temperature controller at a rate of 60 °C per minute. After reaching the desired temperature, the pressure was released to the exact experimental range. The temperature for the high pressure experiments was measured with a C-type thermocouple (W_5_Re_95_-W_26_Re_74_). Cooling water was circulated through a recirculating chiller (CFT-75, Neslab Company, USA) and the temperature of the water was kept at 25 °C. In this study, pressures of 1.0 to 1.5 GPa were conducted in different temperature regimes for a duration of 12 h. The pressure–temperature conditions are tabulated in Table S2.

### Large volume press apparatus

The experiments performed at the highest pressure regime (≥ 2 GPa) were carried out in a high-pressure apparatus of 1000-ton large volume press with the multi-anvil system of 6–8 type Walker module (manufactured by Rockland Research Corporation). In the pressure range of 2–3 GPa, we used the COMPRES 12/18 cell assembly for the experiments, provided by the Department of Chemistry and Biochemistry, Arizona State University. The ram force was set to the expected pressure, calibrated against the Bi-metal phase transitions at 2.55 and 7.7 GPa. Then the temperature was raised at a rate of 50 °C per minute. After the soaking time, the temperature was quenched by terminating the power, and the pressure was brought down at a constant rate. The temperature was monitored with a C-type thermocouple. In this study, the experiments were conducted in the large volume press in different temperature regimes for durations of 12 or 24 h. The pressure–temperature conditions are tabulated in S2.

### Electron micro-probe analysis

After the high pressure and temperature experiments, the resultant samples were first inspected by the electron microscope, using scanning electron microscopy (SEM) and energy dispersive spectrometry (EDS), in the Department of Earth Science of National Cheng Kung University. The observed mineral phases were orthopyroxene, clinopyroxene, Fe-Ti oxides, plagioclase, omphacite, Al_2_Si_2_O_5_ (kyanite), and a SiO_2_ phase. At and below 2 GPa, we also observed the existence of glass in the samples. The chemical compositions of the minerals from the initial basaltic rock and molten glass were quantified by electron micro-probe analysis in the Institute of Earth Sciences, Academia Sinica (Table S3).

Phase identification was carried out by an electron probe micro analyzer (JEOL EPMA JXA-8900R) equipped with four wave-length dispersive spectrometers (WDS). Secondary- and back-scattered electron images were used to guide the analysis on target positions of minerals. A 1 µm spot beam was used for quantitative analysis at an acceleration voltage of 15 kV with a beam current of 12nA. The measured X-ray intensities were corrected by ZAF method using the standard calibration of synthetic chemical-known standard minerals with various diffracting crystals. The standard minerals analyzed for this study are as follows: wollastonite for Si with TAP crystal, rutile for Ti with PET crystal, corundum for Al (TAP), hematite for Fe with LiF crystal, Mn-oxide for Mn (PET), periclase for Mg (TAP), wollastonite for Ca (PET), albite for Na (TAP), and adularia for K(PET). Peak counting for each element and both upper and lower baseline X-rays lasted 10 s and 5 s, respectively. Standards run as unknowns yielded relative standard deviations of < 1% for Si, Na and K, and < 0.5% for other elements. Detection limits were less than 600 ppm for all elements.

## Supplementary Information

Supplementary Table S1.

Supplementary Table S2.

Supplementary Table S3.

## Data Availability

All data generated during this study are included in the supporting information.
