# Supplementary material for: The formation of tonalitic and granodioritic melt from Venusian basalt
Source: Sci Rep. 2022 Jan 31;12:1652. doi: 10.1038/s41598-022-05745-3 (PMC8803830; doi:10.1038/s41598-022-05745-3)
Supplement: Supplementary file 1 — Supplementary Table S1. [file 41598_2022_5745_MOESM1_ESM.docx]

**Table S1. The chemical composition of the starting material of this study, Venera 14 basalt, Archean tholeiite, and MORB.**

| **Sample** | **Starting** | **Venera 14** | **DAT** | **EAT** | **N-MORB**  **(mean)** | **E-MORB**  **(mean)** |
| --- | --- | --- | --- | --- | --- | --- |
| SiO_2_ (wt%) | 51.36 ± 0.83 | 48.7 ± 3.6 | 50.90 | 50.91 | 50.42 ± 0.08 | 50.58 ± 0.33 |
| TiO_2_ | 1.44 ± 0.09 | 1.25 ± 0.41 | 0.95 | 1.53 | 1.53 ± 0.04 | 1.53 ± 0.11 |
| Al_2_O_3_ | 16.34 ± 0.18 | 17.9 ± 2.6 | 15.72 | 15.63 | 15.13 ± 0.12 | 14.94 ± 0.38 |
| FeOt | 8.89 ± 0.43 | 8.8 ± 1.8 | 10.30 | 12.02 | 9.81 ± 0.15 | 9.64 ± 0.48 |
| MnO | 0.18 ± 0.04 | 0.16 ± 0.08 | 0.22 | 0.19 | 0.17 ± 0.004 | 0.16 ± 0.013 |
| MgO | 7.51 ± 0.20 | 8.1 ± 3.3 | 7.64 | 7.01 | 7.76 ± 0.09 | 7.37 ± 0.27 |
| CaO | 11.10 ± 0.16 | 10.3 ± 1.2 | 11.76 | 9.04 | 11.35 ± 0.08 | 11.18 ± 0.27 |
| Na_2_O | 2.94 ± 0.06 | 2.4 ± 0.4* | 2.18 | 2.78 | 2.83 ± 0.05 | 2.72 ± 0.18 |
| K_2_O | 0.24 ± 0.02 | 0.2 ± 0.07 | 0.22 | 0.71 | 0.14 ± 0.11 | 0.39 ± 0.075 |
| P_2_O_5_ |  |  | 0.10 | 0.17 | 0.16 ± 0.004 | 0.24 ± 0.051 |

The Venera 14 data reported at 1σ uncertainty and the Na_2_O* content is calculated^1^. Average Archean tholeiitic compositions from Condie^2^. DAT = depleted Archean tholeiite; enriched Archean tholeiite. The mean (2σ) N-MORB (normal mid-ocean ridge basalt) and E-MORB (enriched mid-ocean ridge basalt) compositions are from Gale et al.^3^

**References**

1. Surkov, Y. A., Barsukov, V. L., Moskalyeva, L. P., Kharyukova, V. P. & Kemurdzhian, A. L. New data on the composition, structure, and properties of Venus rock obtained by Venera 13 and 14. *J. Geophys. Res*. **89** Supp. B393-B402 (1984).
2. Condie, K. C. in *Archean Greenstone Belts* (ed Condie, K. C.) 67-130 (Developments in Precambrian Geology 3, 1981).
3. Gale, A., Dalton, C. A., Langmuir, C. H., Su, Y. & Schilling, J.-G. The mean composition of ocean ridge basalts. *Geochem. Geophys. Geosys*. **14**, 489-518 (2013).
