# Supplementary material for: The formation of tonalitic and granodioritic melt from Venusian basalt
Source: Sci Rep. 2022 Jan 31;12:1652. doi: 10.1038/s41598-022-05745-3 (PMC8803830; doi:10.1038/s41598-022-05745-3)
Supplement: Supplementary file 2 — Supplementary Table S2. [file 41598_2022_5745_MOESM2_ESM.docx]

**Table S2. The pressure-temperature conditions and the mineral phases of this study.**

|  | **P**  **(GPa)** | **T**  **(°C)** | **Duration**  **(hrs)** |  | **Residual Minerals** | | | |  | **Group** |
| --- | --- | --- | --- | --- | --- | --- | --- | --- | --- | --- |
| *Piston cylinder* | 1.0 | 920 | 12 | Opx | Cpx | Ox | Plag |  |  | UM |
|  | 1.5 | 960 | 12 | Opx | Cpx | Ox | Plag |  |  | UM |
|  | 1.5 | 1090 | 12 | Opx | Cpx | Ox | Plag | Qtz | Gl | PM |
|  |  |  |  |  |  |  |  |  |  |  |
| *Large volume press* | 2.0 | 960 | 24 | Opx | Cpx | Ox | Plag | Qtz |  | UM |
|  | 2.0 | 1080 | 24 | Opx | Cpx | Ox | Plag | Qtz | Gl | PM |
|  | 2.0 | 1285 | 24 | Opx | Cpx | Ox | Plag | Qtz | Gl | PM |
|  | 2.5 | 1105 | 24 | Opx | Cpx | Ox | Plag | Qtz |  | UM |
|  | 2.5 | 1195 | 24 | Opx | Cpx | Ox | Qtz | Om | Kya | M |
|  | 3.0 | 1260 | 24 | Opx | Cpx | Ox | Qtz | Om | Kya | M |
|  | 3.0 | 1285 | 24 | Opx | Cpx | Ox | Qtz | Om | Kya | M |

Opx = orthopyroxene; Cpx = clinopyroxene; Ox = Fe-Ti oxide mineral; Qtz = quartz; Plag = plagioclase; Om = omphacite; Kya = kyanite; Gl = glass. UM = unmodified, PM = partial melting, M = metamorphic.
