# Supplementary material for: The formation of tonalitic and granodioritic melt from Venusian basalt
Source: Sci Rep. 2022 Jan 31;12:1652. doi: 10.1038/s41598-022-05745-3 (PMC8803830; doi:10.1038/s41598-022-05745-3)
Supplement: Supplementary file 3 — Supplementary Table S3. [file 41598_2022_5745_MOESM3_ESM.docx]

**The formation of tonalitic and granodioritic melt from Venusian basalt**

Yao Jui Wang^1^, J. Gregory Shellnutt^2*^, Jennifer Kung^1*^, Yoshiyuki Iizuka^3^ & Yu-Ming Lai^2^

^1^National Cheng Kung University, Department of Earth Science, No. 1 University Road, Tainan 701, Taiwan

^2^National Taiwan Normal University, Department of Earth Sciences, 88 Tingzhou Road Section 4, Taipei 11677, Taiwan

^3^Academia Sinica, Institute of Earth Sciences, 128 Academia Road Section 2, Taipei 11529, Taiwan

^*^email: jgshelln@ntnu.edu.tw; jkung@mail.ncku.edu.tw

**Data Repository**

**Supplementary Table S3.** This table consists of electron probe microanalyzer (EPMA) data of orthopyroxene, clinopyroxene, and plagioclase from the initial (pre-melting) synthetic Venera 14 source rock, the glass compositions derived by partial melting during the experiments, and the orthopyroxene, clinopyroxene, and plagioclase of the residual solid. The normative mineral compositions of the glass compositions are calculated assuming and Fe^3+^/Total Fe ratio of 0.3. The pyroxene compositions (rock and residual solid) based on cations of Ca, Mg, and Fe and Mg# are shown as Wo = wollastonite, En = enstatite, Fs = ferrosilite, Mg# = [Mg^2+^/(Mg^2+^+Fe^2+^)]*100. The feldspar compositions (rocks and residual solid) based on Ca, Na, and K cations are shown as An (anorthite) = [Ca^2+^/(Ca^2+^+Na^+^+K^+^)]*100, Ab (albite) = [Na^+^/(Ca^2+^+Na^+^+K^+^)]*100, Or (orthoclase) = [K^+^/(Ca^2+^+Na^+^+K^+^)]*100.

| **Orthopyroxene** | **Rock** |  |  |  |  |  |  |  |
| --- | --- | --- | --- | --- | --- | --- | --- | --- |
| SiO_2_ (wt%) | 55.70 | 55.34 | 54.65 | 54.98 | 55.09 | 54.80 | 54.74 | 54.66 |
| TiO_2_ | 0.32 | 0.24 | 0.30 | 0.27 | 0.30 | 0.21 | 0.34 | 0.41 |
| Al_2_O_3_ | 2.66 | 3.38 | 6.44 | 2.85 | 3.60 | 2.97 | 3.28 | 5.07 |
| FeO | 3.94 | 4.40 | 4.27 | 4.33 | 4.20 | 4.67 | 4.57 | 4.28 |
| MnO | 0.37 | 0.29 | 0.29 | 0.32 | 0.37 | 0.34 | 0.36 | 0.37 |
| MgO | 34.93 | 34.03 | 29.75 | 35.04 | 33.62 | 34.82 | 34.64 | 31.40 |
| CaO | 1.89 | 2.13 | 3.42 | 1.81 | 2.39 | 2.03 | 1.99 | 3.19 |
| Na_2_O | 0.06 | 0.04 | 0.49 | 0.04 | 0.20 | 0.07 | 0.07 | 0.35 |
| K_2_O | 0.01 |  | 0.01 |  |  |  |  | 0.01 |
| Total | 99.88 | 99.85 | 99.62 | 99.64 | 99.77 | 99.91 | 99.99 | 99.74 |
|  |  |  |  |  |  |  |  |  |
| Structural formula based on 6 oxygen | | |  |  |  |  |  |  |
| Si | 1.917 | 1.908 | 1.890 | 1.901 | 1.903 | 1.895 | 1.890 | 1.892 |
| Ti | 0.008 | 0.006 | 0.008 | 0.007 | 0.008 | 0.005 | 0.009 | 0.011 |
| Al | 0.108 | 0.137 | 0.263 | 0.116 | 0.147 | 0.121 | 0.133 | 0.207 |
| Fe | 0.113 | 0.127 | 0.124 | 0.125 | 0.121 | 0.135 | 0.132 | 0.124 |
| Mn | 0.011 | 0.008 | 0.008 | 0.009 | 0.010 | 0.010 | 0.010 | 0.011 |
| Mg | 1.792 | 1.749 | 1.534 | 1.806 | 1.731 | 1.795 | 1.783 | 1.620 |
| Ca | 0.070 | 0.079 | 0.127 | 0.067 | 0.088 | 0.075 | 0.074 | 0.118 |
| Na | 0.004 | 0.003 | 0.033 | 0.003 | 0.013 | 0.005 | 0.005 | 0.032 |
| K |  |  |  |  |  |  |  |  |
|  |  |  |  |  |  |  |  |  |
| Wo | 3.5 | 4.0 | 7.1 | 3.4 | 4.5 | 3.7 | 3.7 | 6.3 |
| En | 90.7 | 89.5 | 85.9 | 90.4 | 89.2 | 89.5 | 89.6 | 87.0 |
| Fs | 5.7 | 6.5 | 6.9 | 6.3 | 6.2 | 6.7 | 6.6 | 6.7 |
| Mg# | 94.1 | 93.2 | 92.5 | 93.5 | 93.5 | 93.0 | 93.1 | 92.9 |

| **Orthopyroxene** | **Rock** |  |  |  |  |  |  |  |
| --- | --- | --- | --- | --- | --- | --- | --- | --- |
| 54.53 | 55.42 | 55.37 | 56.47 | 54.88 | 55.55 | 55.51 | 55.31 | 55.03 |
| 0.28 | 0.25 | 0.26 | 0.27 | 0.23 | 0.24 | 0.25 | 0.46 | 0.42 |
| 3.04 | 3.11 | 2.86 | 2.35 | 6.25 | 2.90 | 2.60 | 3.35 | 6.51 |
| 4.79 | 4.33 | 4.58 | 3.76 | 4.53 | 4.03 | 3.56 | 4.64 | 4.71 |
| 0.31 | 0.41 | 0.33 | 0.35 | 0.21 | 0.33 | 0.38 | 0.48 | 0.35 |
| 34.79 | 34.05 | 34.71 | 34.82 | 30.10 | 34.58 | 35.10 | 32.91 | 27.97 |
| 1.86 | 2.09 | 2.34 | 2.20 | 3.40 | 2.11 | 2.13 | 2.13 | 4.58 |
| 0.02 | 0.11 | 0.06 | 0.01 | 0.39 | 0.09 | 0.08 | 0.11 | 0.56 |
|  |  |  | 0.02 |  | 0.01 | 0.02 | 0.06 | 0.05 |
| 99.62 | 99.77 | 100.51 | 100.25 | 99.99 | 99.84 | 99.63 | 99.45 | 100.18 |
|  |  |  |  |  |  |  |  |  |
|  |  |  |  |  |  |  |  |  |
| 1.891 | 1.913 | 1.903 | 1.934 | 1.892 | 1.914 | 1.914 | 1.918 | 1.907 |
| 0.007 | 0.006 | 0.007 | 0.007 | 0.006 | 0.006 | 0.006 | 0.012 | 0.011 |
| 0.124 | 0.127 | 0.116 | 0.095 | 0.254 | 0.118 | 0.106 | 0.137 | 0.266 |
| 0.139 | 0.125 | 0.132 | 0.108 | 0.131 | 0.116 | 0.103 | 0.135 | 0.137 |
| 0.009 | 0.012 | 0.010 | 0.010 | 0.006 | 0.010 | 0.011 | 0.014 | 0.010 |
| 1.799 | 1.753 | 1.778 | 1.777 | 1.547 | 1.776 | 1.805 | 1.702 | 1.445 |
| 0.069 | 0.077 | 0.086 | 0.081 | 0.126 | 0.078 | 0.079 | 0.079 | 0.170 |
| 0.001 | 0.007 | 0.004 | 0.001 | 0.026 | 0.006 | 0.005 | 0.007 | 0.004 |
|  |  |  | 0.001 |  | 0.000 | 0.001 | 0.003 | 0.002 |
|  |  |  |  |  |  |  |  |  |
| 3.4 | 3.9 | 4.3 | 4.1 | 7.0 | 4.0 | 4.0 | 4.1 | 9.7 |
| 89.6 | 89.7 | 89.1 | 90.4 | 85.8 | 90.2 | 90.8 | 88.8 | 82.5 |
| 6.9 | 6.4 | 6.6 | 5.5 | 7.3 | 5.9 | 5.2 | 7.0 | 7.8 |
| 92.8 | 93.3 | 93.1 | 94.3 | 92.2 | 93.9 | 94.6 | 92.7 | 91.3 |

| **Orthopyroxene** | **Rock** |  |
| --- | --- | --- |
| 54.98 | 54.92 | 54.21 |
| 0.31 | 0.16 | 0.38 |
| 4.06 | 3.85 | 4.55 |
| 4.05 | 4.02 | 4.31 |
| 0.35 | 0.39 | 0.36 |
| 32.94 | 33.38 | 32.28 |
| 2.70 | 2.36 | 2.80 |
| 0.22 | 0.17 | 0.24 |
| 0.01 |  |  |
| 99.62 | 99.25 | 99.13 |
|  |  |  |
|  |  |  |
| 1.901 | 1.904 | 1.888 |
| 0.008 | 0.004 | 0.010 |
| 0.165 | 0.157 | 0.187 |
| 0.117 | 0.117 | 0.126 |
| 0.010 | 0.011 | 0.010 |
| 1.698 | 1.726 | 1.676 |
| 0.100 | 0.088 | 0.104 |
| 0.015 | 0.011 | 0.016 |
| 0.000 | 0.000 |  |
|  |  |  |
| 5.2 | 4.6 | 5.5 |
| 88.7 | 89.4 | 87.9 |
| 6.1 | 6.1 | 6.6 |
| 93.6 | 93.7 | 93.0 |

| **Clinopyroxene** | **Rock** |  |  |  |  |  |  |  |
| --- | --- | --- | --- | --- | --- | --- | --- | --- |
| SiO_2_ (wt%) | 50.48 | 50.58 | 50.67 | 50.88 | 50.38 | 49.97 | 50.34 | 51.26 |
| TiO_2_ | 0.77 | 0.59 | 0.67 | 1.49 | 0.84 | 0.68 | 0.59 | 0.56 |
| Al_2_O_3_ | 5.73 | 5.85 | 5.63 | 6.09 | 4.24 | 4.51 | 10.20 | 6.24 |
| FeO | 6.68 | 6.71 | 6.41 | 8.66 | 6.35 | 6.21 | 6.43 | 5.93 |
| MnO | 0.29 | 0.25 | 0.30 | 0.25 | 0.25 | 0.24 | 0.25 | 0.23 |
| MgO | 17.38 | 16.40 | 16.90 | 13.96 | 17.37 | 16.99 | 13.51 | 16.10 |
| CaO | 17.52 | 18.73 | 18.45 | 16.79 | 19.56 | 20.02 | 17.68 | 19.10 |
| Na_2_O | 0.62 | 0.65 | 0.68 | 0.94 | 0.47 | 0.56 | 1.18 | 0.79 |
| K_2_O | 0.01 | 0.04 |  | 0.18 | 0.05 | 0.02 | 0.04 | 0.01 |
| Total | 99.48 | 99.8 | 99.71 | 99.24 | 99.51 | 99.2 | 100.22 | 100.22 |
|  |  |  |  |  |  |  |  |  |
| Structural formula based on 6 oxygen | | |  |  |  |  |  |  |
| Si | 1.854 | 1.857 | 1.859 | 1.884 | 1.861 | 1.855 | 1.826 | 1.866 |
| Ti | 0.021 | 0.016 | 0.018 | 0.041 | 0.023 | 0.019 | 0.016 | 0.015 |
| Al | 0.248 | 0.253 | 0.243 | 0.266 | 0.185 | 0.197 | 0.436 | 0.268 |
| Fe | 0.205 | 0.206 | 0.197 | 0.268 | 0.196 | 0.193 | 0.195 | 0.181 |
| Mn | 0.009 | 0.008 | 0.009 | 0.008 | 0.008 | 0.008 | 0.008 | 0.007 |
| Mg | 0.952 | 0.898 | 0.924 | 0.771 | 0.957 | 0.940 | 0.730 | 0.874 |
| Ca | 0.689 | 0.737 | 0.725 | 0.666 | 0.774 | 0.796 | 0.687 | 0.745 |
| Na | 0.044 | 0.046 | 0.048 | 0.067 | 0.034 | 0.040 | 0.083 | 0.056 |
| K | 0.000 | 0.002 |  | 0.009 | 0.002 | 0.001 | 0.002 | 0.000 |
|  |  |  |  |  |  |  |  |  |
| Wo | 37.3 | 40.0 | 39.3 | 39.1 | 40.2 | 41.3 | 42.6 | 41.4 |
| En | 51.6 | 48.8 | 50.1 | 45.2 | 49.7 | 48.7 | 45.3 | 48.6 |
| Fs | 11.1 | 11.2 | 10.7 | 15.7 | 10.2 | 10.0 | 12.1 | 10.1 |
| Mg# | 82.3 | 81.3 | 82.4 | 74.2 | 83.0 | 83.0 | 78.9 | 82.8 |

| **Clinopyroxene** | **Rock** |  |  |  |  |  |  |  |
| --- | --- | --- | --- | --- | --- | --- | --- | --- |
| 50.55 | 49.94 | 50.42 | 50.39 | 49.95 | 49.88 | 50.23 | 50.46 | 50.03 |
| 0.62 | 0.68 | 0.78 | 1.12 | 0.61 | 0.59 | 0.91 | 0.60 | 1.22 |
| 5.98 | 3.95 | 5.11 | 6.35 | 4.27 | 4.58 | 5.88 | 4.17 | 6.55 |
| 6.54 | 6.17 | 7.01 | 7.63 | 6.26 | 6.63 | 7.93 | 6.45 | 7.87 |
| 0.26 | 0.31 | 0.36 | 0.29 | 0.26 | 0.30 | 0.31 | 0.29 | 0.27 |
| 16.16 | 17.75 | 16.49 | 16.74 | 17.02 | 17.24 | 15.69 | 17.66 | 14.29 |
| 18.84 | 19.90 | 18.81 | 16.67 | 20.27 | 19.73 | 17.89 | 19.91 | 18.17 |
| 0.60 | 0.41 | 0.59 | 0.55 | 0.46 | 0.42 | 0.69 | 0.40 | 0.64 |
| 0.04 |  |  | 0.05 |  |  | 0.03 |  | 0.09 |
| 99.59 | 99.11 | 99.57 | 99.79 | 99.1 | 99.37 | 99.56 | 99.94 | 99.13 |
|  |  |  |  |  |  |  |  |  |
|  |  |  |  |  |  |  |  |  |
| 1.859 | 1.855 | 1.861 | 1.847 | 1.857 | 1.850 | 1.856 | 1.859 | 1.856 |
| 0.017 | 0.019 | 0.022 | 0.031 | 0.017 | 0.016 | 0.025 | 0.017 | 0.034 |
| 0.256 | 0.173 | 0.222 | 0.274 | 0.187 | 0.200 | 0.256 | 0.181 | 0.286 |
| 0.201 | 0.192 | 0.216 | 0.234 | 0.195 | 0.206 | 0.245 | 0.199 | 0.244 |
| 0.008 | 0.010 | 0.011 | 0.009 | 0.008 | 0.009 | 0.010 | 0.009 | 0.008 |
| 0.886 | 0.983 | 0.908 | 0.915 | 0.944 | 0.953 | 0.864 | 0.970 | 0.790 |
| 0.742 | 0.792 | 0.744 | 0.655 | 0.808 | 0.784 | 0.708 | 0.786 | 0.722 |
| 0.043 | 0.030 | 0.042 | 0.039 | 0.033 | 0.030 | 0.049 | 0.029 | 0.046 |
| 0.002 |  |  | 0.002 |  |  | 0.001 |  | 0.004 |
|  |  |  |  |  |  |  |  |  |
| 40.6 | 40.3 | 39.8 | 36.3 | 41.5 | 40.3 | 39.0 | 40.2 | 41.1 |
| 48.4 | 50.0 | 48.6 | 50.7 | 48.5 | 49.0 | 47.6 | 49.6 | 45.0 |
| 11.0 | 9.8 | 11.6 | 13.0 | 10.0 | 10.6 | 13.5 | 10.2 | 13.9 |
| 81.5 | 83.7 | 80.8 | 79.6 | 82.9 | 82.2 | 77.9 | 83.0 | 76.4 |

| **Clinopyroxene** | **Rock** |  |  |  |  |  |  |  |
| --- | --- | --- | --- | --- | --- | --- | --- | --- |
| 50.26 | 49.45 | 50.53 | 48.56 | 49.34 | 50.16 | 50.18 | 50.09 | 51.40 |
| 0.70 | 0.87 | 0.37 | 1.05 | 0.71 | 0.53 | 0.64 | 0.93 | 1.26 |
| 3.95 | 6.55 | 11.35 | 7.16 | 5.59 | 6.68 | 3.98 | 11.74 | 7.76 |
| 6.52 | 7.00 | 5.33 | 7.47 | 6.83 | 6.07 | 6.11 | 7.33 | 7.80 |
| 0.32 | 0.23 | 0.21 | 0.37 | 0.20 | 0.29 | 0.26 | 0.22 | 0.30 |
| 18.18 | 15.49 | 12.61 | 15.30 | 16.12 | 16.18 | 17.55 | 11.19 | 13.19 |
| 19.04 | 19.16 | 17.88 | 18.50 | 19.71 | 18.81 | 20.16 | 16.04 | 16.30 |
| 0.39 | 0.72 | 1.15 | 0.83 | 0.57 | 0.63 | 0.45 | 1.80 | 1.44 |
| 0.01 |  |  | 0.02 | 0.02 | 0.01 | 0.02 | 0.04 | 0.21 |
| 99.37 | 99.47 | 99.43 | 99.26 | 99.09 | 99.36 | 99.35 | 99.38 | 99.66 |
|  |  |  |  |  |  |  |  |  |
|  |  |  |  |  |  |  |  |  |
| 1.860 | 1.830 | 1.833 | 1.806 | 1.836 | 1.845 | 1.860 | 1.829 | 1.882 |
| 0.019 | 0.024 | 0.010 | 0.029 | 0.020 | 0.015 | 0.018 | 0.026 | 0.035 |
| 0.172 | 0.286 | 0.485 | 0.314 | 0.245 | 0.290 | 0.174 | 0.505 | 0.335 |
| 0.202 | 0.217 | 0.162 | 0.232 | 0.213 | 0.187 | 0.189 | 0.224 | 0.239 |
| 0.010 | 0.007 | 0.006 | 0.012 | 0.006 | 0.009 | 0.008 | 0.007 | 0.009 |
| 1.003 | 0.854 | 0.682 | 0.848 | 0.894 | 0.887 | 0.970 | 0.609 | 0.720 |
| 0.755 | 0.760 | 0.695 | 0.737 | 0.786 | 0.741 | 0.801 | 0.628 | 0.640 |
| 0.028 | 0.052 | 0.081 | 0.060 | 0.041 | 0.045 | 0.032 | 0.127 | 0.102 |
| 0.000 |  |  | 0.001 | 0.001 | 0.000 | 0.001 | 0.002 | 0.010 |
|  |  |  |  |  |  |  |  |  |
| 38.5 | 41.5 | 45.2 | 40.6 | 41.5 | 40.8 | 40.9 | 43.0 | 40.0 |
| 51.2 | 46.6 | 44.3 | 46.7 | 47.2 | 48.9 | 49.5 | 41.7 | 45.0 |
| 10.3 | 11.9 | 10.5 | 12.8 | 11.3 | 10.3 | 9.6 | 15.3 | 14.9 |
| 83.2 | 79.7 | 80.8 | 78.5 | 80.8 | 82.6 | 83.7 | 73.1 | 75.1 |

| **Clinopyroxene** | **Rock** |  |  |  |  |  |  |  |
| --- | --- | --- | --- | --- | --- | --- | --- | --- |
| 50.27 | 52.07 | 50.18 | 49.74 | 49.33 | 51.08 | 51.30 | 49.50 | 51.12 |
| 0.79 | 0.94 | 1.04 | 1.02 | 0.44 | 0.76 | 0.86 | 0.54 | 0.91 |
| 4.73 | 5.32 | 5.47 | 8.97 | 10.87 | 5.66 | 5.00 | 13.19 | 5.66 |
| 6.43 | 6.58 | 7.75 | 7.30 | 5.73 | 6.66 | 6.52 | 5.39 | 7.04 |
| 0.33 | 0.32 | 0.26 | 0.15 | 0.19 | 0.28 | 0.30 | 0.22 | 0.29 |
| 17.91 | 20.13 | 17.45 | 12.52 | 12.41 | 16.55 | 17.06 | 10.55 | 15.29 |
| 18.18 | 13.70 | 17.18 | 18.18 | 19.12 | 17.91 | 17.95 | 18.47 | 18.66 |
| 0.50 | 0.52 | 0.47 | 1.56 | 1.36 | 0.96 | 0.68 | 1.73 | 0.60 |
| 0.05 | 0.10 | 0.07 | 0.10 |  | 0.05 | 0.13 | 0.02 | 0.10 |
| 99.19 | 99.68 | 99.87 | 99.54 | 99.45 | 99.91 | 99.8 | 99.61 | 99.67 |
|  |  |  |  |  |  |  |  |  |
|  |  |  |  |  |  |  |  |  |
| 1.857 | 1.882 | 1.845 | 1.833 | 1.807 | 1.869 | 1.879 | 1.800 | 1.880 |
| 0.022 | 0.026 | 0.029 | 0.028 | 0.012 | 0.021 | 0.024 | 0.015 | 0.025 |
| 0.206 | 0.227 | 0.237 | 0.390 | 0.469 | 0.244 | 0.216 | 0.565 | 0.245 |
| 0.199 | 0.199 | 0.238 | 0.225 | 0.176 | 0.204 | 0.200 | 0.164 | 0.216 |
| 0.010 | 0.010 | 0.008 | 0.005 | 0.006 | 0.009 | 0.009 | 0.007 | 0.009 |
| 0.986 | 1.085 | 0.956 | 0.688 | 0.678 | 0.903 | 0.931 | 0.572 | 0.838 |
| 0.719 | 0.531 | 0.677 | 0.718 | 0.750 | 0.702 | 0.704 | 0.720 | 0.735 |
| 0.036 | 0.036 | 0.034 | 0.111 | 0.097 | 0.068 | 0.048 | 0.122 | 0.043 |
| 0.002 | 0.005 | 0.003 | 0.005 |  | 0.002 | 0.006 | 0.001 | 0.005 |
|  |  |  |  |  |  |  |  |  |
| 37.8 | 29.3 | 36.2 | 44.0 | 46.8 | 38.8 | 38.4 | 49.5 | 41.1 |
| 51.8 | 59.8 | 51.1 | 42.2 | 42.3 | 49.9 | 50.7 | 39.3 | 46.8 |
| 10.5 | 11.0 | 12.7 | 13.8 | 11.0 | 11.3 | 10.9 | 11.3 | 12.1 |
| 83.2 | 84.5 | 80.1 | 75.4 | 79.4 | 81.6 | 82.3 | 77.7 | 79.5 |

| **Clinopyroxene** | **Rock** |  |  |  |  |  |  |  |  |
| --- | --- | --- | --- | --- | --- | --- | --- | --- | --- |
| 51.04 | 50.33 | 51.39 | 51.39 | 49.61 | 52.03 | 49.63 | 49.60 | 49.63 | 51.08 |
| 0.68 | 0.36 | 1.24 | 0.85 | 1.07 | 1.12 | 0.68 | 0.66 | 1.41 | 0.78 |
| 4.63 | 8.39 | 6.86 | 6.09 | 5.64 | 6.86 | 9.06 | 7.18 | 7.41 | 5.20 |
| 6.46 | 5.72 | 7.44 | 6.80 | 7.70 | 6.52 | 6.42 | 6.67 | 9.05 | 6.35 |
| 0.42 | 0.24 | 0.23 | 0.28 | 0.35 | 0.25 | 0.22 | 0.24 | 0.33 | 0.29 |
| 19.68 | 14.86 | 14.21 | 15.14 | 15.78 | 14.63 | 13.46 | 15.31 | 15.17 | 16.63 |
| 15.95 | 19.47 | 17.13 | 17.81 | 19.06 | 16.42 | 18.64 | 19.70 | 15.46 | 18.89 |
| 0.56 | 0.96 | 1.14 | 1.01 | 0.60 | 1.05 | 1.08 | 0.81 | 0.72 | 0.65 |
| 0.04 | 0.02 | 0.16 | 0.10 | 0.06 | 0.20 | 0.07 | 0.03 | 0.11 | 0.04 |
| 99.46 | 100.35 | 99.80 | 99.47 | 99.87 | 99.08 | 99.26 | 100.2 | 99.29 | 99.91 |
|  |  |  |  |  |  |  |  |  |  |
|  |  |  |  |  |  |  |  |  |  |
| 1.867 | 1.830 | 1.881 | 1.886 | 1.836 | 1.902 | 1.827 | 1.821 | 1.835 | 1.871 |
| 0.019 | 0.010 | 0.034 | 0.023 | 0.030 | 0.031 | 0.019 | 0.018 | 0.039 | 0.021 |
| 0.200 | 0.360 | 0.296 | 0.263 | 0.246 | 0.296 | 0.393 | 0.311 | 0.323 | 0.225 |
| 0.198 | 0.174 | 0.228 | 0.209 | 0.238 | 0.199 | 0.198 | 0.205 | 0.280 | 0.195 |
| 0.013 | 0.007 | 0.007 | 0.009 | 0.010 | 0.008 | 0.007 | 0.007 | 0.010 | 0.009 |
| 1.073 | 0.806 | 0.775 | 0.829 | 0.871 | 0.798 | 0.739 | 0.838 | 0.836 | 0.908 |
| 0.625 | 0.759 | 0.672 | 0.701 | 0.756 | 0.643 | 0.735 | 0.775 | 0.612 | 0.742 |
| 0.040 | 0.068 | 0.081 | 0.072 | 0.043 | 0.074 | 0.077 | 0.058 | 0.052 | 0.046 |
| 0.002 | 0.001 | 0.007 | 0.005 | 0.003 | 0.009 | 0.003 | 0.001 | 0.005 | 0.003 |
|  |  |  |  |  |  |  |  |  |  |
| 33.0 | 43.6 | 40.1 | 40.3 | 40.5 | 39.2 | 44.0 | 42.6 | 35.4 | 40.2 |
| 56.6 | 46.3 | 46.3 | 47.7 | 46.7 | 48.7 | 44.2 | 46.1 | 48.4 | 49.2 |
| 10.4 | 10.0 | 13.6 | 12.0 | 12.8 | 12.1 | 11.8 | 11.3 | 16.2 | 10.6 |
| 84.4 | 82.2 | 77.3 | 79.9 | 78.5 | 80.0 | 78.9 | 80.3 | 74.9 | 82.3 |

| **Plagioclase** | **Rock** |  |  |  |  |  |  |  |
| --- | --- | --- | --- | --- | --- | --- | --- | --- |
| SiO_2_ (wt%) | 51.43 | 50.96 | 50.80 | 53.62 | 50.75 | 51.07 | 51.13 | 51.79 |
| TiO_2_ | 0.19 | 0.00 | 0.15 | 0.98 | 0.10 | 0.05 | 0.18 | 0.09 |
| Al_2_O_3_ | 27.30 | 28.46 | 26.67 | 25.00 | 28.51 | 28.45 | 28.00 | 27.53 |
| FeO | 2.60 | 2.64 | 2.78 | 4.04 | 2.84 | 2.75 | 2.82 | 2.76 |
| MnO |  |  | 0.06 | 0.10 | 0.06 | 0.03 |  | 0.02 |
| MgO | 0.61 | 0.30 | 1.52 | 0.82 | 0.42 | 0.39 | 0.43 | 0.51 |
| CaO | 13.58 | 13.77 | 14.00 | 12.15 | 13.79 | 14.21 | 13.72 | 13.71 |
| Na_2_O | 3.21 | 3.07 | 3.09 | 3.07 | 3.26 | 3.11 | 3.43 | 3.30 |
| K_2_O | 0.04 | 0.07 | 0.06 | 0.19 | 0.08 | 0.05 | 0.07 | 0.08 |
| Total | 98.96 | 99.27 | 99.13 | 99.97 | 99.81 | 100.11 | 99.78 | 99.79 |
|  |  |  |  |  |  |  |  |  |
| Structural formula based on 8 oxygen | | | |  |  |  |  |  |
| Si | 2.390 | 2.361 | 2.368 | 2.469 | 2.346 | 2.352 | 2.363 | 2.389 |
| Ti | 0.007 |  | 0.005 | 0.034 | 0.003 | 0.002 | 0.006 | 0.003 |
| Al | 1.495 | 1.554 | 1.465 | 1.357 | 1.553 | 1.544 | 1.525 | 1.497 |
| Fe | 0.101 | 0.102 | 0.108 | 0.156 | 0.110 | 0.106 | 0.109 | 0.106 |
| Mn |  |  | 0.002 | 0.004 | 0.002 | 0.001 |  | 0.001 |
| Mg | 0.042 | 0.021 | 0.106 | 0.056 | 0.029 | 0.027 | 0.030 | 0.035 |
| Ca | 0.676 | 0.684 | 0.699 | 0.600 | 0.683 | 0.701 | 0.679 | 0.678 |
| Na | 0.289 | 0.276 | 0.279 | 0.274 | 0.292 | 0.278 | 0.306 | 0.295 |
| K | 0.002 | 0.004 | 0.004 | 0.010 | 0.005 | 0.003 | 0.004 | 0.005 |
|  |  |  |  |  |  |  |  |  |
| An | 69.9 | 71.0 | 71.2 | 67.9 | 69.7 | 71.4 | 68.7 | 69.3 |
| Ab | 29.9 | 28.6 | 28.4 | 31.0 | 29.8 | 28.3 | 30.9 | 30.2 |
| Or | 0.2 | 0.4 | 0.4 | 1.1 | 0.5 | 0.3 | 0.4 | 0.5 |

| **Plagioclase** | **Rock** |  |  |  |  |  |
| --- | --- | --- | --- | --- | --- | --- |
| 51.35 | 50.31 | 50.20 | 50.54 | 50.73 | 51.61 | 52.63 |
| 0.33 | 0.12 | 0.09 | 0.16 | 0.15 | 0.40 | 0.50 |
| 27.13 | 28.49 | 27.88 | 28.28 | 28.47 | 26.60 | 25.06 |
| 3.27 | 2.69 | 2.80 | 2.78 | 2.68 | 3.33 | 3.86 |
| 0.09 | 0.04 |  |  |  | 0.01 | 0.06 |
| 0.87 | 0.34 | 0.47 | 0.42 | 0.40 | 0.94 | 1.03 |
| 13.84 | 14.01 | 13.99 | 14.17 | 14.15 | 13.48 | 12.62 |
| 2.91 | 3.11 | 3.19 | 3.02 | 3.13 | 3.13 | 3.27 |
| 0.09 | 0.05 | 0.06 | 0.07 | 0.04 | 0.09 | 0.14 |
| 99.88 | 99.16 | 98.68 | 99.44 | 99.75 | 99.59 | 99.17 |
|  |  |  |  |  |  |  |
|  |  |  |  |  |  |  |
| 2.375 | 2.340 | 2.349 | 2.345 | 2.345 | 2.392 | 2.449 |
| 0.011 | 0.004 | 0.003 | 0.006 | 0.005 | 0.014 | 0.018 |
| 1.479 | 1.562 | 1.537 | 1.547 | 1.551 | 1.453 | 1.374 |
| 0.126 | 0.105 | 0.110 | 0.108 | 0.104 | 0.129 | 0.150 |
| 0.004 | 0.002 |  |  |  |  | 0.002 |
| 0.060 | 0.024 | 0.033 | 0.029 | 0.028 | 0.065 | 0.071 |
| 0.686 | 0.698 | 0.701 | 0.704 | 0.701 | 0.670 | 0.629 |
| 0.261 | 0.280 | 0.289 | 0.272 | 0.281 | 0.281 | 0.295 |
| 0.005 | 0.003 | 0.004 | 0.004 | 0.002 | 0.005 | 0.008 |
|  |  |  |  |  |  |  |
| 72.1 | 71.2 | 70.5 | 71.8 | 71.2 | 70.1 | 67.5 |
| 27.4 | 28.5 | 29.1 | 27.8 | 28.6 | 29.4 | 31.7 |
| 0.5 | 0.3 | 0.4 | 0.4 | 0.2 | 0.5 | 0.9 |

| **Glass** |  |  |  |  |  |
| --- | --- | --- | --- | --- | --- |
| **Temperature (^o^C)** | **1090** | **1090** | **1090** | ***Average*** | ***Uncertainty*** |
| **Pressure (GPa)** | **1.5** | **1.5** | **1.5** |  |  |
| SiO_2_ (wt%) | 66.55 | 64.87 | 64.51 | ***65.3*** | ***1.0*** |
| TiO_2_ | 0.47 | 0.79 | 0.67 | ***0.6*** | ***0.2*** |
| Al_2_O_3_ | 17.94 | 19.28 | 19.34 | ***18.9*** | ***0.7*** |
| FeOt | 3.09 | 3.56 | 2.88 | ***3.2*** | ***0.3*** |
| MnO |  | 0.01 | 0.09 | ***0.1*** | ***0.0*** |
| MgO | 0.96 | 0.26 | 1.11 | ***0.8*** | ***0.4*** |
| CaO | 7.53 | 7.45 | 7.73 | ***7.6*** | ***0.1*** |
| Na_2_O | 3.10 | 3.47 | 3.27 | ***3.3*** | ***0.2*** |
| K_2_O | 0.32 | 0.29 | 0.37 | ***0.3*** | ***0.0*** |
| Total | 99.96 | 99.98 | 99.97 | ***100.0*** |  |
|  |  |  |  |  |  |
| Mg# | 35.6 | 11.5 | 40.7 | ***30.4*** |  |
|  |  |  |  |  |  |
| Normative Minerology (vol%) | |  |  |  |  |
| Quartz | 31.15 | 28.14 | 26.85 | ***28.5*** |  |
| Anorthite | 33.85 | 35.92 | 36.72 | ***35.6*** |  |
| Albite | 27.49 | 30.64 | 28.99 | ***29.2*** |  |
| Orthoclase | 2.03 | 1.84 | 2.34 | ***1.9*** |  |
| Corundum |  |  |  |  |  |
| Diopside | 2.18 | 0.52 | 0.92 | ***1.2*** |  |
| Hypersthene | 1.47 | 0.56 | 2.20 | ***1.6*** |  |
| Wollastonite |  |  |  |  |  |
| Rutile |  |  |  |  |  |
| Ilmenite | 0.52 | 0.87 | 0.74 | ***0.7*** |  |
| Magnetite | 1.31 | 1.51 | 1.22 | ***1.4*** |  |
| Hematite |  |  |  |  |  |

| **Glass** |  |  |  |  |  |  |
| --- | --- | --- | --- | --- | --- | --- |
| **1080** | **1080** | **1080** | **1080** | **1080** | **1080** | **1080** |
| **2.0** | **2.0** | **2.0** | **2.0** | **2.0** | **2.0** | **2.0** |
| 74.67 | 64.99 | 69.34 | 75.56 | 75.39 | 76.52 | 76.14 |
| 0.22 | 0.14 | 0.83 | 0.37 | 0.42 | 0.89 | 0.45 |
| 14.17 | 20.32 | 16.62 | 14.31 | 14.52 | 14.91 | 14.32 |
| 1.17 | 1.78 | 5.09 | 0.54 | 2.72 | 0.83 | 2.63 |
| 0.06 |  | 0.44 | 0.80 |  | 0.61 |  |
| 0.71 | 0.23 | 0.96 | 2.16 | 1.08 | 0.67 | 1.00 |
| 6.08 | 8.66 | 4.13 | 3.74 | 3.27 | 2.94 | 2.99 |
| 2.79 | 3.73 | 1.71 | 1.57 | 1.58 | 1.59 | 1.62 |
| 0.15 | 0.16 | 0.88 | 0.95 | 1.02 | 1.03 | 0.85 |
| 100.02 | 100.01 | 100 | 100 | 100 | 99.99 | 100 |
|  |  |  |  |  |  |  |
| 52.0 | 18.7 | 25.2 | 87.7 | 41.4 | 59.0 | 40.4 |
|  |  |  |  |  |  |  |
|  |  |  |  |  |  |  |
| 45.53 | 25.12 | 47.57 | 52.88 | 55.51 | 58.04 | 57.56 |
| 25.18 | 37.59 | 20.86 | 18.54 | 16.22 | 14.52 | 14.84 |
| 24.39 | 32.74 | 15.56 | 13.96 | 14.08 | 14.11 | 14.44 |
| 0.94 | 1.00 | 5.72 | 6.02 | 6.50 | 6.53 | 5.42 |
|  |  | 3.76 | 2.69 | 3.36 | 4.02 | 3.66 |
| 2.92 | 1.09 |  |  |  |  |  |
| 0.32 |  | 3.38 | 5.39 | 2.70 | 1.45 | 2.46 |
|  | 0.76 |  |  |  |  |  |
|  |  |  |  |  |  |  |
| 0.24 | 0.15 | 0.93 | 0.41 | 0.46 | 0.98 | 0.50 |
| 0.49 | 0.75 | 2.21 | 0.23 | 1.16 | 0.35 | 1.12 |
|  |  |  |  |  |  |  |

| **Glass** |  |  |  |  |  |  |
| --- | --- | --- | --- | --- | --- | --- |
| **1080** | **1080** | **1080** | **1080** | ***Average*** | ***Uncertainty*** | **1285** |
| **2.0** | **2.0** | **2.0** | **2.0** |  |  | **2.0** |
| 75.90 | 70.93 | 75.02 | 74.35 | ***73.5*** | ***5.8*** | 72.60 |
| 0.44 | 0.47 | 0.30 | 0.52 | ***0.5*** | ***0.4*** | 0.41 |
| 14.58 | 17.83 | 14.80 | 15.24 | ***15.6*** | ***3.1*** | 15.85 |
| 2.61 | 1.83 | 2.76 | 2.38 | ***2.2*** | ***2.3*** | 1.66 |
|  | 0.04 |  | 0.24 | ***0.4*** | ***0.4*** | 0.08 |
| 1.03 | 0.59 | 1.36 | 1.11 | ***1.0*** | ***1.0*** | 0.73 |
| 2.54 | 4.93 | 3.10 | 3.45 | ***4.2*** | ***3.1*** | 3.78 |
| 1.59 | 3.07 | 1.52 | 1.78 | ***2.1*** | ***1.1*** | 3.18 |
| 1.31 | 0.30 | 1.13 | 0.93 | ***0.8*** | ***0.6*** | 1.70 |
| 100 | 99.99 | 99.99 | 100 | ***100.2*** |  | 99.99 |
|  |  |  |  |  |  |  |
| 41.3 | 36.5 | 46.8 | 45.4 | ***44.4*** |  | 43.9 |
|  |  |  |  |  |  |  |
|  |  |  |  |  |  |  |
| 56.63 | 41.68 | 55.04 | 53.13 | ***48.9*** |  | 39.06 |
| 12.61 | 24.26 | 15.42 | 17.13 | ***20.7*** |  | 18.39 |
| 14.18 | 27.14 | 13.58 | 15.88 | ***18.6*** |  | 27.80 |
| 8.35 | 1.90 | 7.22 | 5.93 | ***5.1*** |  | 10.62 |
| 4.10 | 2.39 | 3.77 | 3.48 | ***2.5*** |  | 1.29 |
|  |  |  |  |  |  |  |
| 2.53 | 1.34 | 3.47 | 2.86 | ***2.8*** |  | 1.70 |
|  |  |  |  |  |  |  |
|  |  |  |  |  |  |  |
| 0.49 | 0.51 | 0.33 | 0.57 | ***0.6*** |  | 0.44 |
| 1.12 | 0.78 | 1.18 | 1.02 | ***0.9*** |  | 0.69 |
|  |  |  |  |  |  |  |

| **Glass** |  |  |  |  |  |  |
| --- | --- | --- | --- | --- | --- | --- |
| **1285** | **1285** | **1285** | **1285** | **1285** | **1285** | **1285** |
| **2.0** | **2.0** | **2.0** | **2.0** | **2.0** | **2.0** | **2.0** |
| 72.35 | 69.42 | 68.79 | 74.88 | 75.51 | 75.29 | 75.15 |
| 0.51 | 0.38 | 0.15 | 0.47 | 0.47 | 0.46 | 0.58 |
| 15.81 | 18.00 | 18.95 | 14.49 | 14.08 | 14.41 | 14.40 |
| 1.75 | 1.64 | 0.77 | 1.34 | 1.48 | 1.36 | 1.44 |
| 0.05 | 0.09 | 0.04 | 0.14 | 0.06 | 0.09 | 0.06 |
| 0.89 | 0.87 | 0.07 | 0.91 | 0.72 | 0.79 | 0.82 |
| 3.96 | 5.27 | 7.10 | 2.64 | 2.60 | 2.59 | 2.75 |
| 2.91 | 2.91 | 3.96 | 2.63 | 2.62 | 2.45 | 2.42 |
| 1.77 | 1.42 | 0.15 | 2.50 | 2.46 | 2.55 | 2.38 |
| 100 | 100 | 99.98 | 100 | 100 | 99.99 | 100 |
|  |  |  |  |  |  |  |
| 47.5 | 48.6 | 13.9 | 54.8 | 46.4 | 50.9 | 50.4 |
|  |  |  |  |  |  |  |
|  |  |  |  |  |  |  |
| 39.65 | 35.19 | 30.61 | 43.76 | 44.96 | 45.39 | 45.76 |
| 19.30 | 25.77 | 32.65 | 12.83 | 12.62 | 12.59 | 13.38 |
| 25.49 | 25.57 | 34.42 | 22.96 | 22.84 | 21.39 | 21.16 |
| 11.08 | 8.92 | 0.93 | 15.60 | 15.33 | 15.91 | 14.87 |
| 1.29 | 1.42 |  | 1.80 | 1.61 | 1.97 | 1.93 |
|  |  | 0.56 |  |  |  |  |
| 1.91 | 2.04 |  | 1.98 | 1.52 | 1.68 | 1.72 |
|  |  | 0.35 |  |  |  |  |
|  |  |  |  |  |  |  |
| 0.55 | 0.41 | 0.16 | 0.51 | 0.51 | 0.50 | 0.63 |
| 0.73 | 0.69 | 0.32 | 0.56 | 0.62 | 0.57 | 0.43 |
|  |  |  |  |  |  | 0.12 |

| **Glass** |  |  |  |  |  |  |
| --- | --- | --- | --- | --- | --- | --- |
| **1285** | **1285** | **1285** | **1285** | **1285** | **1285** | **1285** |
| **2.0** | **2.0** | **2.0** | **2.0** | **2.0** | **2.0** | **2.0** |
| 73.21 | 72.80 | 75.68 | 75.44 | 74.27 | 65.33 | 72.30 |
| 0.47 | 0.36 | 0.41 | 0.45 | 0.53 | 0.22 | 0.31 |
| 15.55 | 15.70 | 14.07 | 14.34 | 14.80 | 20.99 | 16.45 |
| 1.46 | 1.48 | 1.52 | 1.47 | 1.52 | 1.37 | 1.41 |
| 0.09 | 0.07 | 0.11 | 0.14 | 0.13 | 0.05 | 0.01 |
| 0.87 | 0.71 | 0.76 | 0.81 | 0.91 | 0.39 | 0.66 |
| 3.34 | 3.54 | 2.65 | 2.75 | 3.13 | 6.42 | 4.16 |
| 2.78 | 3.02 | 2.43 | 2.42 | 2.62 | 4.17 | 2.82 |
| 2.23 | 2.32 | 2.39 | 2.18 | 2.08 | 1.06 | 1.89 |
| 100 | 100 | 100.02 | 100 | 99.99 | 100 | 100.01 |
|  |  |  |  |  |  |  |
| 51.5 | 46.1 | 47.1 | 49.6 | 51.6 | 33.7 | 45.5 |
|  |  |  |  |  |  |  |
|  |  |  |  |  |  |  |
| 40.83 | 38.32 | 46.34 | 46.81 | 43.90 | 22.95 | 39.44 |
| 16.25 | 17.17 | 12.88 | 13.40 | 15.25 | 31.23 | 20.23 |
| 24.31 | 26.31 | 21.23 | 21.18 | 22.94 | 36.45 | 24.64 |
| 13.94 | 14.45 | 14.92 | 13.64 | 13.02 | 6.62 | 11.80 |
| 1.69 | 1.20 | 1.81 | 2.04 | 1.73 | 0.88 | 1.49 |
|  |  |  |  |  |  |  |
| 1.87 | 1.64 | 1.75 | 1.83 | 1.93 | 1.06 | 1.49 |
|  |  |  |  |  |  |  |
|  |  |  |  |  |  |  |
| 0.51 | 0.39 | 0.44 | 0.49 | 0.57 | 0.24 | 0.34 |
| 0.61 | 0.62 | 0.63 | 0.62 | 0.63 | 0.57 | 0.59 |
|  |  |  |  |  |  |  |

| **Glass** |  |  |  |  |  |  |
| --- | --- | --- | --- | --- | --- | --- |
| **1285** | **1285** | **1285** | **1285** | **1285** | **1285** | **1285** |
| **2.0** | **2.0** | **2.0** | **2.0** | **2.0** | **2.0** | **2.0** |
| 74.80 | 74.58 | 75.59 | 74.20 | 76.35 | 73.49 | 74.01 |
| 0.37 | 0.83 | 0.57 | 0.50 | 0.31 | 0.34 | 0.44 |
| 14.86 | 15.38 | 15.02 | 15.23 | 15.86 | 16.10 | 15.28 |
| 1.53 | 2.08 | 1.57 | 2.12 | 0.16 | 2.61 | 0.98 |
| 0.10 | 0.02 |  | 0.56 | 0.13 |  | 0.23 |
| 0.91 | 1.12 | 0.97 | 1.28 | 1.06 | 0.88 | 3.57 |
| 2.89 | 3.23 | 3.15 | 2.93 | 3.15 | 3.26 | 3.03 |
| 2.33 | 1.83 | 2.00 | 2.05 | 1.80 | 2.03 | 1.31 |
| 2.20 | 0.93 | 1.13 | 1.13 | 1.18 | 1.29 | 1.17 |
| 99.99 | 100 | 100 | 100 | 100 | 100 | 100.02 |
|  |  |  |  |  |  |  |
| 51.5 | 49.0 | 52.4 | 51.8 | 92.2 | 37.5 | 86.7 |
|  |  |  |  |  |  |  |
|  |  |  |  |  |  |  |
| 46.21 | 53.85 | 53.09 | 51.23 | 54.61 | 49.99 | 52.43 |
| 14.11 | 16.03 | 15.51 | 14.55 | 15.45 | 16.16 | 15.14 |
| 20.44 | 16.32 | 17.69 | 18.29 | 15.87 | 18.08 | 11.77 |
| 13.79 | 5.93 | 7.14 | 7.21 | 7.43 | 8.21 | 7.51 |
| 2.31 | 3.79 | 3.28 | 3.67 | 4.04 | 3.75 | 4.43 |
|  |  |  |  |  |  |  |
| 2.10 | 2.40 | 2.06 | 3.58 | 2.24 | 2.32 | 7.81 |
|  |  |  |  |  |  |  |
|  |  |  |  | 0.05 |  |  |
| 0.40 | 0.92 | 0.62 | 0.55 | 0.26 | 0.37 | 0.49 |
| 0.64 | 0.54 | 0.45 | 0.91 |  | 1.11 | 0.42 |
|  | 0.24 | 0.14 |  | 0.05 |  |  |

| **Glass** |  |  |  |  |  |
| --- | --- | --- | --- | --- | --- |
| **1285** | **1285** | **1285** | **1285** | ***Average*** | ***Uncertainty*** |
| **2.0** | **2.0** | **2.0** | **2.0** |  |  |
| 74.17 | 75.57 | 75.89 | 75.89 | ***73.8*** | ***5.5*** |
| 0.19 | 0.19 | 0.16 | 0.77 | ***0.4*** | ***0.3*** |
| 15.30 | 15.38 | 15.27 | 15.54 | ***15.7*** | ***3.5*** |
| 2.53 | 1.72 | 1.37 | 0.16 | ***1.5*** | ***1.2*** |
| 0.08 | 0.10 |  | 0.66 | ***0.1*** | ***0.3*** |
| 1.30 | 0.98 | 1.17 | 1.00 | ***1.0*** | ***1.8*** |
| 3.03 | 2.71 | 3.19 | 2.86 | ***3.5*** | ***2.3*** |
| 1.99 | 2.07 | 1.56 | 1.85 | ***2.5*** | ***1.4*** |
| 1.41 | 1.29 | 1.39 | 1.28 | ***1.7*** | ***1.2*** |
| 100 | 100.01 | 100 | 100.01 | ***100.0*** |  |
|  |  |  |  |  |  |
| 47.8 | 50.4 | 60.4 | 91.8 | ***53.8*** |  |
|  |  |  |  |  |  |
|  |  |  |  |  |  |
| 50.12 | 52.75 | 54.49 | 54.34 | ***44.7*** |  |
| 15.00 | 13.37 | 15.72 | 14.08 | ***17.1*** |  |
| 17.71 | 18.34 | 13.81 | 16.36 | ***21.9*** |  |
| 8.97 | 8.17 | 8.79 | 8.09 | ***10.7*** |  |
| 3.44 | 3.88 | 3.71 | 4.07 | ***2.3*** |  |
|  |  |  |  |  |  |
| 3.48 | 2.55 | 2.74 | 2.14 | ***2.2*** |  |
|  |  |  |  |  |  |
|  |  |  |  |  |  |
| 0.21 | 0.21 | 0.18 | 0.84 | ***0.4*** |  |
| 1.08 | 0.73 | 0.58 | 0.07 | ***0.6*** |  |
|  |  |  |  |  |  |

| **Orthopyroxene** | **Residual** |  |  |  |  |  |  |  |
| --- | --- | --- | --- | --- | --- | --- | --- | --- |
| **Temperature (^o^C)** | **1090** | **1090** | **1090** | **1090** | **1090** | **1090** | **1090** | **1090** |
| **Pressure (GPa)** | **1.5** | **1.5** | **1.5** | **1.5** | **1.5** | **1.5** | **1.5** | **1.5** |
| SiO_2_ (wt%) | 54.25 | 55.31 | 55.68 | 54.24 | 55.44 | 55.52 | 54.88 | 55.00 |
| TiO_2_ | 0.25 | 0.33 | 0.33 | 0.37 | 0.19 | 0.27 | 0.27 | 0.31 |
| Al_2_O_3_ | 3.83 | 2.38 | 2.34 | 3.14 | 2.58 | 2.85 | 3.68 | 4.24 |
| FeO | 5.95 | 4.30 | 4.18 | 4.85 | 4.17 | 4.15 | 4.62 | 4.67 |
| MnO | 0.46 | 0.34 | 0.40 | 0.30 | 0.34 | 0.35 | 0.37 | 0.31 |
| MgO | 32.77 | 34.40 | 35.49 | 33.86 | 34.40 | 34.67 | 34.18 | 33.56 |
| CaO | 1.57 | 1.93 | 1.99 | 2.47 | 1.91 | 1.87 | 2.44 | 2.54 |
| Na_2_O | 0.03 | 0.03 | 0.05 | 0.06 | 0.04 | 0.11 | 0.13 | 0.16 |
| K_2_O |  |  | 0.03 |  |  | 0.02 | 0.02 |  |
| Total | 99.11 | 99.02 | 100.49 | 99.29 | 99.07 | 99.81 | 100.59 | 100.79 |
|  |  |  |  |  |  |  |  |  |
| Structural formula based on 6 oxygen | | |  |  |  |  |  |  |
| Si | 1.897 | 1.923 | 1.910 | 1.891 | 1.924 | 1.914 | 1.886 | 1.885 |
| Ti | 0.007 | 0.009 | 0.009 | 0.010 | 0.005 | 0.007 | 0.007 | 0.008 |
| Al | 0.158 | 0.098 | 0.095 | 0.129 | 0.106 | 0.116 | 0.149 | 0.171 |
| Fe | 0.174 | 0.125 | 0.120 | 0.141 | 0.121 | 0.120 | 0.133 | 0.134 |
| Mn | 0.014 | 0.010 | 0.012 | 0.009 | 0.010 | 0.010 | 0.011 | 0.009 |
| Mg | 1.708 | 1.783 | 1.815 | 1.760 | 1.780 | 1.782 | 1.752 | 1.715 |
| Ca | 0.059 | 0.072 | 0.073 | 0.092 | 0.071 | 0.069 | 0.090 | 0.093 |
| Na | 0.002 | 0.002 | 0.003 | 0.004 | 0.003 | 0.007 | 0.009 | 0.011 |
| K |  |  | 0.001 |  |  | 0.001 | 0.001 |  |
|  |  |  |  |  |  |  |  |  |
| Wo | 3.0 | 3.6 | 3.6 | 4.6 | 3.6 | 3.5 | 4.6 | 4.8 |
| En | 88.0 | 90.1 | 90.4 | 88.3 | 90.3 | 90.4 | 88.7 | 88.3 |
| Fs | 9.0 | 6.3 | 6.0 | 7.1 | 6.1 | 6.1 | 6.7 | 6.9 |
| Mg# | 90.8 | 93.4 | 93.8 | 92.6 | 93.6 | 93.7 | 92.9 | 92.8 |

| **Orthopyroxene** | **Residual** |  |  |  |  |  |  |  |
| --- | --- | --- | --- | --- | --- | --- | --- | --- |
| **1090** | **1080** | **1080** | **1080** | **1080** | **1080** | **1080** | **1080** | **1285** |
| **1.5** | **2.0** | **2.0** | **2.0** | **2.0** | **2.0** | **2.0** | **2.0** | **2.0** |
| 54.36 | 54.37 | 52.57 | 54.20 | 53.30 | 51.92 | 53.48 | 52.22 | 51.02 |
| 0.26 | 0.24 | 0.20 | 0.23 | 0.30 | 0.27 | 0.22 | 0.21 | 0.39 |
| 3.36 | 2.97 | 3.58 | 2.72 | 2.88 | 5.95 | 2.93 | 5.41 | 6.64 |
| 5.33 | 6.21 | 7.82 | 4.52 | 4.88 | 4.60 | 5.66 | 5.35 | 10.86 |
| 0.33 | 0.39 | 0.37 | 0.35 | 0.37 | 0.31 | 0.32 | 0.36 | 0.50 |
| 33.91 | 33.78 | 32.17 | 34.50 | 34.53 | 30.32 | 33.47 | 31.67 | 28.58 |
| 2.19 | 1.81 | 2.04 | 1.86 | 2.10 | 4.51 | 2.02 | 2.66 | 1.95 |
| 0.06 | 0.10 | 0.09 | 0.03 | 0.04 | 0.39 | 0.05 | 0.30 | 0.13 |
| 0.01 |  | 0.01 | 0.03 | 0.02 | 0.02 | 0.03 |  |  |
| 99.81 | 99.87 | 98.85 | 98.44 | 98.42 | 98.29 | 98.18 | 98.18 | 100.07 |
|  |  |  |  |  |  |  |  |  |
|  |  |  |  |  |  |  |  |  |
| 1.888 | 1.894 | 1.868 | 1.901 | 1.878 | 1.840 | 1.892 | 1.849 | 1.815 |
| 0.007 | 0.006 | 0.005 | 0.006 | 0.008 | 0.007 | 0.006 | 0.006 | 0.010 |
| 0.138 | 0.122 | 0.150 | 0.112 | 0.120 | 0.249 | 0.122 | 0.226 | 0.278 |
| 0.155 | 0.181 | 0.232 | 0.133 | 0.144 | 0.136 | 0.167 | 0.158 | 0.323 |
| 0.010 | 0.012 | 0.011 | 0.010 | 0.011 | 0.009 | 0.010 | 0.011 | 0.015 |
| 1.756 | 1.754 | 1.704 | 1.804 | 1.814 | 1.602 | 1.765 | 1.672 | 1.515 |
| 0.081 | 0.068 | 0.078 | 0.070 | 0.079 | 0.171 | 0.077 | 0.101 | 0.074 |
| 0.004 | 0.007 | 0.006 | 0.002 | 0.003 | 0.027 | 0.003 | 0.021 | 0.009 |
| 0.000 |  | 0.000 | 0.001 | 0.001 | 0.001 | 0.001 |  |  |
|  |  |  |  |  |  |  |  |  |
| 4.1 | 3.4 | 3.9 | 3.5 | 3.9 | 9.0 | 3.8 | 5.2 | 3.9 |
| 88.2 | 87.6 | 84.6 | 89.9 | 89.1 | 83.9 | 87.9 | 86.6 | 79.2 |
| 7.8 | 9.0 | 11.5 | 6.6 | 7.1 | 7.1 | 8.3 | 8.2 | 16.9 |
| 91.9 | 90.6 | 88.0 | 93.1 | 92.6 | 92.2 | 91.4 | 91.4 | 82.4 |

| **Orthopyroxene** | **Residual** |  |  |  |  |  |  |  |
| --- | --- | --- | --- | --- | --- | --- | --- | --- |
| **1285** | **1285** | **1285** | **1285** | **1285** | **1285** | **1285** | **1285** | **1285** |
| **2.0** | **2.0** | **2.0** | **2.0** | **2.0** | **2.0** | **2.0** | **2.0** | **2.0** |
| 49.71 | 54.72 | 53.62 | 50.77 | 48.55 | 50.48 | 51.7 | 51.36 | 48.25 |
| 0.45 | 0.19 | 0.33 | 0.36 | 0.31 | 0.38 | 0.26 | 0.31 | 0.69 |
| 7.37 | 4.26 | 4.34 | 7.04 | 9.28 | 7.95 | 5.27 | 7.13 | 8.8 |
| 11.45 | 9.32 | 9.68 | 10.42 | 12.35 | 10.28 | 10.03 | 10.51 | 12.53 |
| 0.35 | 0.70 | 0.54 | 0.44 | 0.35 | 0.34 | 0.45 | 0.44 | 0.42 |
| 28.41 | 29.79 | 30.23 | 28.22 | 27.34 | 27.76 | 29.68 | 28.42 | 27.69 |
| 1.76 | 1.43 | 1.72 | 1.65 | 1.95 | 2 | 1.73 | 2.42 | 1.66 |
| 0.13 | 0.16 | 0.12 | 0.1 | 0.08 | 0.22 | 0.13 | 0.27 | 0.11 |
|  | 0.01 | 0.01 |  |  |  | 0.01 | 0.01 | 0.01 |
| 99.63 | 100.58 | 100.59 | 99.00 | 100.21 | 99.41 | 99.26 | 100.87 | 100.16 |
|  |  |  |  |  |  |  |  |  |
|  |  |  |  |  |  |  |  |  |
| 1.782 | 1.912 | 1.881 | 1.818 | 1.739 | 1.801 | 1.845 | 1.811 | 1.732 |
| 0.012 | 0.005 | 0.009 | 0.010 | 0.008 | 0.010 | 0.007 | 0.008 | 0.019 |
| 0.311 | 0.175 | 0.179 | 0.297 | 0.392 | 0.334 | 0.222 | 0.296 | 0.372 |
| 0.343 | 0.272 | 0.284 | 0.312 | 0.370 | 0.307 | 0.299 | 0.310 | 0.376 |
| 0.011 | 0.021 | 0.016 | 0.013 | 0.011 | 0.010 | 0.014 | 0.013 | 0.013 |
| 1.518 | 1.552 | 1.581 | 1.507 | 1.460 | 1.476 | 1.579 | 1.494 | 1.482 |
| 0.068 | 0.054 | 0.065 | 0.063 | 0.075 | 0.076 | 0.066 | 0.091 | 0.064 |
| 0.009 | 0.011 | 0.008 | 0.007 | 0.006 | 0.015 | 0.009 | 0.018 | 0.008 |
|  |  | 0.000 |  |  |  | 0.000 | 0.000 | 0.000 |
|  |  |  |  |  |  |  |  |  |
| 3.5 | 2.9 | 3.4 | 3.3 | 3.9 | 4.1 | 3.4 | 4.8 | 3.3 |
| 78.7 | 82.6 | 81.9 | 80.1 | 76.6 | 79.4 | 81.2 | 78.8 | 77.1 |
| 17.8 | 14.5 | 14.7 | 16.6 | 19.4 | 16.5 | 15.4 | 16.4 | 19.6 |
| 81.6 | 85.1 | 84.8 | 82.8 | 79.8 | 82.8 | 84.1 | 82.8 | 79.8 |

| **Orthopyroxene** | **Residual** |  |
| --- | --- | --- |
| **1285** | **1285** | **1285** |
| **2.0** | **2.0** | **2.0** |
| 51.06 | 50.28 | 49.28 |
| 0.42 | 0.36 | 0.39 |
| 6.47 | 7.85 | 8.00 |
| 10.45 | 11.22 | 11.48 |
| 0.49 | 0.42 | 0.41 |
| 28.7 | 28.43 | 28.21 |
| 1.76 | 1.82 | 1.95 |
| 0.12 | 0.09 | 0.15 |
|  |  |  |
| 99.47 | 100.47 | 99.87 |
|  |  |  |
|  |  |  |
| 1.822 | 1.784 | 1.765 |
| 0.011 | 0.010 | 0.011 |
| 0.272 | 0.328 | 0.338 |
| 0.312 | 0.333 | 0.344 |
| 0.015 | 0.013 | 0.012 |
| 1.527 | 1.504 | 1.506 |
| 0.067 | 0.069 | 0.075 |
| 0.008 | 0.006 | 0.010 |
|  |  |  |
|  |  |  |
| 3.5 | 3.6 | 3.9 |
| 80.1 | 78.9 | 78.2 |
| 16.4 | 17.5 | 17.9 |
| 83.0 | 81.9 | 81.4 |

| **Clinopyroxene** | **Residual** |  |  |  |  |  |  |  |
| --- | --- | --- | --- | --- | --- | --- | --- | --- |
| **Temperature (^o^C)** | **1090** | **1090** | **1090** | **1090** | **1090** | **1090** | **1090** | **1090** |
| **Pressure (GPa)** | **1.5** | **1.5** | **1.5** | **1.5** | **1.5** | **1.5** | **1.5** | **1.5** |
| SiO_2_ (wt%) | 48.88 | 49.85 | 51.19 | 50.40 | 49.64 | 49.37 | 49.00 | 48.46 |
| TiO_2_ | 0.78 | 0.78 | 0.50 | 0.63 | 0.68 | 0.83 | 0.74 | 0.84 |
| Al_2_O_3_ | 5.16 | 5.39 | 3.64 | 3.79 | 3.88 | 6.84 | 4.61 | 5.85 |
| FeO | 7.68 | 7.73 | 6.42 | 6.13 | 7.07 | 7.39 | 7.42 | 8.50 |
| MnO | 0.33 | 0.32 | 0.27 | 0.27 | 0.41 | 0.35 | 0.33 | 0.30 |
| MgO | 16.36 | 15.85 | 17.93 | 18.38 | 18.81 | 14.79 | 17.13 | 15.27 |
| CaO | 19.86 | 18.90 | 18.87 | 19.33 | 18.89 | 18.66 | 19.38 | 19.12 |
| Na_2_O | 0.71 | 0.66 | 0.47 | 0.32 | 0.27 | 0.98 | 0.39 | 0.79 |
| K_2_O | 0.01 | 0.02 | 0.01 | 0.01 |  |  |  | 0.03 |
| Total | 99.77 | 99.5 | 99.3 | 99.26 | 99.65 | 99.21 | 99 | 99.16 |
|  |  |  |  |  |  |  |  |  |
| Structural formula based on 6 oxygen | | |  |  |  |  |  |  |
| Si | 1.820 | 1.850 | 1.889 | 1.864 | 1.839 | 1.834 | 1.833 | 1.818 |
| Ti | 0.022 | 0.022 | 0.014 | 0.018 | 0.019 | 0.023 | 0.021 | 0.024 |
| Al | 0.226 | 0.236 | 0.158 | 0.165 | 0.169 | 0.299 | 0.203 | 0.259 |
| Fe | 0.239 | 0.240 | 0.198 | 0.190 | 0.219 | 0.230 | 0.232 | 0.267 |
| Mn | 0.010 | 0.010 | 0.008 | 0.008 | 0.013 | 0.011 | 0.010 | 0.010 |
| Mg | 0.908 | 0.877 | 0.987 | 1.013 | 1.039 | 0.819 | 0.955 | 0.854 |
| Ca | 0.792 | 0.752 | 0.746 | 0.766 | 0.750 | 0.743 | 0.777 | 0.769 |
| Na | 0.051 | 0.047 | 0.034 | 0.023 | 0.190 | 0.071 | 0.028 | 0.057 |
| K | 0.000 | 0.001 | 0.000 | 0.000 |  |  |  | 0.001 |
|  |  |  |  |  |  |  |  |  |
| Wo | 40.8 | 40.2 | 38.6 | 38.9 | 37.4 | 41.5 | 39.6 | 40.7 |
| En | 46.8 | 46.9 | 51.1 | 51.4 | 51.7 | 45.7 | 48.6 | 45.2 |
| Fs | 12.3 | 12.8 | 10.3 | 9.6 | 10.9 | 12.8 | 11.8 | 14.1 |
| Mg# | 79.2 | 78.5 | 83.3 | 84.2 | 82.6 | 78.1 | 80.5 | 76.2 |

| **Clinopyroxene** | **Residual** |  |  |  |  |  |  |  |
| --- | --- | --- | --- | --- | --- | --- | --- | --- |
| **1080** | **1080** | **1080** | **1080** | **1080** | **1285** | **1285** | **1285** | **1285** |
| **2.0** | **2.0** | **2.0** | **2.0** | **2.0** | **2.0** | **2.0** | **2.0** | **2.0** |
| 46.82 | 48.49 | 48.26 | 48.46 | 46.62 | 44.43 | 44.05 | 45.74 | 47.95 |
| 1.24 | 0.66 | 0.65 | 0.57 | 0.71 | 0.86 | 0.73 | 0.56 | 0.61 |
| 6.82 | 4.12 | 7.48 | 6.65 | 9.69 | 11.88 | 11.59 | 10.56 | 7.06 |
| 9.79 | 7.31 | 8.57 | 8.67 | 10.17 | 10.62 | 10.70 | 10.17 | 7.96 |
| 0.37 | 0.24 | 0.34 | 0.28 | 0.30 | 0.21 | 0.16 | 0.22 | 0.21 |
| 17.58 | 17.51 | 14.91 | 14.86 | 13.04 | 12.24 | 12.09 | 12.97 | 15.65 |
| 15.44 | 20.47 | 17.30 | 18.12 | 17.30 | 17.21 | 18.55 | 18.16 | 18.90 |
| 0.77 | 0.40 | 1.17 | 0.99 | 1.08 | 1.28 | 1.04 | 1.19 | 0.65 |
|  | 0.01 |  | 0.01 | 0.03 | 0.01 |  | 0.02 | 0.01 |
| 98.83 | 99.21 | 98.68 | 98.61 | 98.94 | 98.74 | 98.91 | 99.59 | 99 |
|  |  |  |  |  |  |  |  |  |
|  |  |  |  |  |  |  |  |  |
| 1.764 | 1.818 | 1.809 | 1.822 | 1.757 | 1.686 | 1.676 | 1.719 | 1.794 |
| 0.035 | 0.019 | 0.018 | 0.016 | 0.020 | 0.025 | 0.021 | 0.016 | 0.017 |
| 0.294 | 0.182 | 0.330 | 0.295 | 0.430 | 0.531 | 0.520 | 0.468 | 0.311 |
| 0.309 | 0.229 | 0.269 | 0.273 | 0.320 | 0.337 | 0.341 | 0.320 | 0.249 |
| 0.012 | 0.008 | 0.011 | 0.009 | 0.010 | 0.007 | 0.005 | 0.007 | 0.007 |
| 0.988 | 0.979 | 0.833 | 0.833 | 0.733 | 0.692 | 0.686 | 0.727 | 0.873 |
| 0.623 | 0.822 | 0.695 | 0.730 | 0.698 | 0.700 | 0.756 | 0.731 | 0.758 |
| 0.056 | 0.029 | 0.085 | 0.072 | 0.079 | 0.094 | 0.077 | 0.087 | 0.047 |
|  |  |  | 0.000 | 0.001 | 0.000 |  | 0.001 | 0.000 |
|  |  |  |  |  |  |  |  |  |
| 32.4 | 40.5 | 38.7 | 39.8 | 39.9 | 40.5 | 42.4 | 41.1 | 40.3 |
| 51.5 | 48.2 | 46.4 | 45.4 | 41.9 | 40.0 | 38.5 | 40.9 | 46.4 |
| 16.1 | 11.3 | 15.0 | 14.9 | 18.3 | 19.5 | 19.1 | 18.0 | 13.2 |
| 76.2 | 81.0 | 75.6 | 75.3 | 69.6 | 67.2 | 66.8 | 69.4 | 77.8 |

| **Clinopyroxene** | **Residual** |  |  |  |  |  |  |  |
| --- | --- | --- | --- | --- | --- | --- | --- | --- |
| **1285** | **1285** | **1285** | **1285** | **1285** | **1285** | **1285** | **1285** | **1285** |
| **2.0** | **2.0** | **2.0** | **2.0** | **2.0** | **2.0** | **2.0** | **2.0** | **2.0** |
| 45.33 | 44.21 | 48.39 | 44.24 | 46.49 | 48.02 | 46.41 | 45.71 | 46.50 |
| 0.43 | 0.86 | 0.39 | 0.95 | 0.47 | 0.38 | 0.53 | 0.58 | 0.62 |
| 10.86 | 12.84 | 8.96 | 13.26 | 9.11 | 8.67 | 9.73 | 11.13 | 10.01 |
| 9.83 | 10.58 | 8.61 | 10.97 | 8.61 | 8.59 | 9.07 | 9.35 | 9.48 |
| 0.15 | 0.27 | 0.19 | 0.31 | 0.16 | 0.19 | 0.14 | 0.20 | 0.24 |
| 13.07 | 11.84 | 15.21 | 11.81 | 14.06 | 14.67 | 13.61 | 12.90 | 13.83 |
| 18.97 | 17.61 | 17.06 | 16.99 | 18.36 | 18.47 | 18.00 | 18.63 | 17.35 |
| 1.02 | 1.21 | 1.11 | 1.24 | 1.09 | 0.90 | 1.20 | 1.07 | 1.18 |
|  | 0.03 |  | 0.03 |  |  |  | 0.01 | 0.01 |
| 99.66 | 99.45 | 99.92 | 99.8 | 98.35 | 99.89 | 98.69 | 99.58 | 99.22 |
|  |  |  |  |  |  |  |  |  |
|  |  |  |  |  |  |  |  |  |
| 1.704 | 1.666 | 1.786 | 1.661 | 1.756 | 1.780 | 1.748 | 1.712 | 1.742 |
| 0.012 | 0.024 | 0.011 | 0.027 | 0.013 | 0.011 | 0.015 | 0.016 | 0.017 |
| 0.481 | 0.570 | 0.390 | 0.587 | 0.406 | 0.379 | 0.432 | 0.491 | 0.442 |
| 0.309 | 0.333 | 0.266 | 0.344 | 0.272 | 0.266 | 0.286 | 0.293 | 0.297 |
| 0.005 | 0.009 | 0.006 | 0.010 | 0.005 | 0.006 | 0.004 | 0.006 | 0.008 |
| 0.732 | 0.665 | 0.837 | 0.661 | 0.792 | 0.811 | 0.764 | 0.720 | 0.773 |
| 0.764 | 0.711 | 0.675 | 0.683 | 0.743 | 0.734 | 0.727 | 0.748 | 0.697 |
| 0.074 | 0.088 | 0.079 | 0.090 | 0.080 | 0.065 | 0.088 | 0.078 | 0.086 |
|  | 0.001 |  | 0.001 |  |  |  | 0.000 | 0.000 |
|  |  |  |  |  |  |  |  |  |
| 42.3 | 41.6 | 38.0 | 40.5 | 41.1 | 40.5 | 40.9 | 42.5 | 39.4 |
| 40.6 | 38.9 | 47.1 | 39.2 | 43.8 | 44.8 | 43.0 | 40.9 | 43.7 |
| 17.1 | 19.5 | 15.0 | 20.4 | 15.1 | 14.7 | 16.1 | 16.6 | 16.8 |
| 70.3 | 66.6 | 75.9 | 65.8 | 74.4 | 75.3 | 72.8 | 71.1 | 72.2 |

| **Clinopyroxene** | **Residual** |  |  |  |  |  |
| --- | --- | --- | --- | --- | --- | --- |
| **1285** | **1285** | **1285** | **1285** | **1285** | **1285** | **1285** |
| **2.0** | **2.0** | **2.0** | **2.0** | **2.0** | **2.0** | **2.0** |
| 44.86 | 44.44 | 44.75 | 44.26 | 44.14 | 47.00 | 46.51 |
| 1.20 | 0.72 | 0.67 | 0.71 | 0.84 | 0.37 | 0.57 |
| 9.39 | 12.06 | 11.79 | 11.90 | 12.60 | 9.65 | 9.86 |
| 12.06 | 10.10 | 9.82 | 10.39 | 9.73 | 8.49 | 9.00 |
| 0.36 | 0.19 | 0.15 | 0.20 | 0.22 | 0.17 | 0.11 |
| 13.89 | 11.69 | 12.37 | 11.82 | 12.02 | 14.05 | 13.74 |
| 16.02 | 18.33 | 18.04 | 18.56 | 17.89 | 17.62 | 18.38 |
| 0.80 | 1.08 | 1.13 | 1.13 | 1.15 | 1.16 | 1.13 |
|  |  | 0.04 |  |  | 0.01 |  |
| 98.58 | 98.61 | 98.76 | 98.97 | 98.59 | 98.52 | 99.3 |
|  |  |  |  |  |  |  |
|  |  |  |  |  |  |  |
| 1.713 | 1.687 | 1.693 | 1.680 | 1.673 | 1.764 | 1.742 |
| 0.034 | 0.021 | 0.019 | 0.020 | 0.024 | 0.010 | 0.016 |
| 0.423 | 0.540 | 0.526 | 0.532 | 0.563 | 0.427 | 0.435 |
| 0.385 | 0.321 | 0.311 | 0.330 | 0.308 | 0.267 | 0.282 |
| 0.012 | 0.006 | 0.005 | 0.006 | 0.007 | 0.005 | 0.003 |
| 0.791 | 0.662 | 0.698 | 0.669 | 0.679 | 0.786 | 0.767 |
| 0.655 | 0.746 | 0.731 | 0.755 | 0.726 | 0.709 | 0.738 |
| 0.059 | 0.080 | 0.083 | 0.083 | 0.084 | 0.084 | 0.082 |
|  |  | 0.002 |  |  | 0.000 |  |
|  |  |  |  |  |  |  |
| 35.8 | 43.1 | 42.0 | 43.0 | 42.4 | 40.2 | 41.3 |
| 43.2 | 38.3 | 40.1 | 38.1 | 39.6 | 44.6 | 42.9 |
| 21.0 | 18.6 | 17.9 | 18.8 | 18.0 | 15.2 | 15.8 |
| 67.3 | 67.3 | 69.2 | 67.0 | 68.8 | 74.6 | 73.1 |

| **Plagioclase** | **Residual** |  |  |  |  |  |  |  |
| --- | --- | --- | --- | --- | --- | --- | --- | --- |
| **Temperature (^o^C)** | **1090** | **1090** | **1090** | **1090** | **1090** | **1090** | **1090** | **1090** |
| **Pressure (GPa)** | **1.5** | **1.5** | **1.5** | **1.5** | **1.5** | **1.5** | **1.5** | **1.5** |
| SiO_2_ (wt%) | 51.68 | 52.77 | 55.01 | 54.15 | 53.42 | 51.73 | 51.85 | 52.38 |
| TiO_2_ | 0.22 | 0.18 | 0.18 | 0.13 | 0.86 | 0.07 | 0.09 | 0.19 |
| Al_2_O_3_ | 27.76 | 25.83 | 25.65 | 25.48 | 23.60 | 26.16 | 28.23 | 27.22 |
| FeO | 2.81 | 3.12 | 1.63 | 1.62 | 4.52 | 2.46 | 2.01 | 2.56 |
| MnO | 0.02 | 0.05 | 0.07 | 0.08 | 0.09 | 0.02 |  | 0.02 |
| MgO | 0.25 | 0.68 | 2.01 | 1.93 | 1.11 | 2.06 | 0.32 | 0.39 |
| CaO | 12.96 | 12.19 | 10.52 | 10.65 | 10.96 | 13.57 | 13.14 | 11.60 |
| Na_2_O | 3.67 | 3.73 | 4.30 | 4.05 | 3.64 | 3.00 | 3.56 | 4.07 |
| K_2_O | 0.24 | 0.28 | 0.31 | 0.38 | 0.31 | 0.20 | 0.22 | 0.35 |
| Total | 99.61 | 98.83 | 99.68 | 98.47 | 98.51 | 99.27 | 99.42 | 98.78 |
|  |  |  |  |  |  |  |  |  |
| Structural formula based on 8 oxygen | | |  |  |  |  |  |  |
| Si | 2.388 | 2.454 | 2.505 | 2.498 | 2.502 | 2.339 | 2.390 | 2.429 |
| Ti | 0.008 | 0.006 | 0.006 | 0.005 | 0.030 | 0.002 | 0.003 | 0.007 |
| Al | 1.512 | 1.416 | 1.377 | 1.385 | 1.303 | 1.430 | 1.534 | 1.488 |
| Fe | 0.109 | 0.121 | 0.062 | 0.063 | 0.177 | 0.095 | 0.077 | 0.099 |
| Mn | 0.001 | 0.002 | 0.003 | 0.003 | 0.004 | 0.001 |  | 0.001 |
| Mg | 0.017 | 0.047 | 0.136 | 0.133 | 0.077 | 0.142 | 0.022 | 0.027 |
| Ca | 0.642 | 0.608 | 0.513 | 0.526 | 0.550 | 0.674 | 0.649 | 0.576 |
| Na | 0.329 | 0.336 | 0.380 | 0.362 | 0.331 | 0.270 | 0.318 | 0.366 |
| K | 0.014 | 0.017 | 0.018 | 0.022 | 0.019 | 0.012 | 0.013 | 0.021 |
|  |  |  |  |  |  |  |  |  |
| An | 65.2 | 63.3 | 56.3 | 57.8 | 61.1 | 70.5 | 66.2 | 59.8 |
| Ab | 33.4 | 35.0 | 41.7 | 39.8 | 36.8 | 28.2 | 32.4 | 38.0 |
| Or | 1.4 | 1.8 | 2.0 | 2.4 | 2.1 | 1.3 | 1.3 | 2.2 |

| **Plagioclase** | **Residual** |  |  |  |  |  |  |  |
| --- | --- | --- | --- | --- | --- | --- | --- | --- |
| **1090** | **1090** | **1090** | **1090** | **1090** | **1090** | **1090** | **1090** | **1090** |
| **1.5** | **1.5** | **1.5** | **1.5** | **1.5** | **1.5** | **1.5** | **1.5** | **1.5** |
| 50.31 | 50.18 | 52.11 | 50.72 | 51.79 | 52.10 | 52.14 | 51.20 | 50.84 |
| 0.44 | 0.06 | 0.12 | 0.31 | 0.12 |  | 0.22 | 1.41 | 0.08 |
| 26.01 | 26.67 | 28.03 | 25.39 | 27.88 | 28.76 | 27.29 | 25.35 | 28.36 |
| 3.71 | 3.11 | 2.04 | 2.87 | 2.54 | 1.98 | 1.87 | 5.13 | 2.75 |
| 0.10 | 0.12 | 0.02 | 0.04 | 0.01 | 0.02 | 0.02 | 0.03 | 0.05 |
| 1.40 | 1.30 | 0.32 | 2.17 | 0.23 | 0.24 | 0.87 | 0.46 | 0.26 |
| 13.10 | 13.94 | 12.54 | 13.79 | 13.19 | 12.90 | 12.43 | 10.86 | 13.60 |
| 3.47 | 3.19 | 3.82 | 3.24 | 3.29 | 3.65 | 3.92 | 3.89 | 3.41 |
| 0.21 | 0.19 | 0.29 | 0.22 | 0.28 | 0.25 | 0.30 | 0.28 | 0.21 |
| 98.75 | 98.76 | 99.29 | 98.75 | 99.33 | 99.90 | 99.06 | 98.61 | 99.56 |
|  |  |  |  |  |  |  |  |  |
|  |  |  |  |  |  |  |  |  |
| 2.366 | 2.356 | 2.403 | 2.380 | 2.395 | 2.387 | 2.410 | 2.410 | 2.355 |
| 0.016 | 0.002 | 0.004 | 0.011 | 0.004 |  | 0.008 | 0.050 | 0.003 |
| 1.442 | 1.476 | 1.524 | 1.404 | 1.519 | 1.553 | 1.487 | 1.407 | 1.549 |
| 0.146 | 0.122 | 0.079 | 0.113 | 0.098 | 0.076 | 0.072 | 0.202 | 0.107 |
| 0.004 | 0.005 | 0.001 | 0.002 | 0.000 | 0.001 | 0.001 | 0.001 | 0.002 |
| 0.098 | 0.091 | 0.022 | 0.152 | 0.016 | 0.016 | 0.060 | 0.032 | 0.018 |
| 0.660 | 0.701 | 0.620 | 0.693 | 0.653 | 0.633 | 0.616 | 0.548 | 0.675 |
| 0.316 | 0.290 | 0.342 | 0.295 | 0.295 | 0.324 | 0.351 | 0.355 | 0.306 |
| 0.013 | 0.010 | 0.017 | 0.013 | 0.017 | 0.015 | 0.018 | 0.017 | 0.012 |
|  |  |  |  |  |  |  |  |  |
| 66.7 | 70.0 | 63.3 | 69.2 | 67.7 | 65.1 | 62.5 | 59.6 | 68.0 |
| 32.0 | 29.0 | 34.9 | 29.5 | 30.6 | 33.3 | 35.6 | 38.6 | 30.8 |
| 1.3 | 1.0 | 1.7 | 1.3 | 1.8 | 1.5 | 1.8 | 1.8 | 1.2 |

| **Plagioclase** | **Residual** |  |  |  |  |  |  |  |
| --- | --- | --- | --- | --- | --- | --- | --- | --- |
| **1090** | **1090** | **1090** | **1090** | **1090** | **1080** | **1080** | **1080** | **1080** |
| **1.5** | **1.5** | **1.5** | **1.5** | **1.5** | **2.0** | **2.0** | **2.0** | **2.0** |
| 51.13 | 52.44 | 54.12 | 51.72 | 53.26 | 53.82 | 54.79 | 54.17 | 53.49 |
| 0.19 | 0.00 | 0.34 | 1.47 | 0.23 | 0.14 | 0.15 | 0.17 | 0.12 |
| 28.32 | 27.69 | 25.63 | 25.01 | 27.19 | 27.18 | 27.37 | 27.79 | 28.26 |
| 2.51 | 1.85 | 2.79 | 6.95 | 1.99 | 1.88 | 1.31 | 1.4 | 1.66 |
| 0.04 |  | 0.01 | 0.01 |  | 0.07 | 0.06 | 0.01 | 0.06 |
| 0.20 | 0.44 | 0.83 | 0.50 | 0.27 | 0.90 | 0.15 | 0.25 | 0.25 |
| 13.26 | 12.39 | 10.90 | 9.30 | 11.60 | 11.68 | 11.03 | 11.51 | 11.99 |
| 3.43 | 4.10 | 4.63 | 4.04 | 4.01 | 4.63 | 5.31 | 4.87 | 4.35 |
| 0.20 | 0.27 | 0.32 | 0.39 | 0.33 | 0.19 | 0.18 | 0.16 | 0.15 |
| 99.28 | 99.18 | 99.57 | 99.39 | 98.88 | 100.49 | 100.35 | 100.33 | 100.33 |
|  |  |  |  |  |  |  |  |  |
|  |  |  |  |  |  |  |  |  |
| 2.368 | 2.419 | 2.488 | 2.426 | 2.455 | 2.446 | 2.481 | 2.456 | 2.430 |
| 0.007 |  | 0.012 | 0.052 | 0.008 | 0.005 | 0.005 | 0.006 | 0.004 |
| 1.546 | 1.505 | 1.389 | 1.383 | 1.477 | 1.456 | 1.461 | 1.485 | 1.513 |
| 0.097 | 0.071 | 0.107 | 0.273 | 0.077 | 0.071 | 0.050 | 0.053 | 0.063 |
| 0.002 |  | 0.000 | 0.000 | 0.000 | 0.003 | 0.002 | 0.000 | 0.002 |
| 0.014 | 0.030 | 0.057 | 0.035 | 0.019 | 0.061 | 0.010 | 0.017 | 0.017 |
| 0.658 | 0.612 | 0.537 | 0.467 | 0.573 | 0.569 | 0.535 | 0.559 | 0.584 |
| 0.308 | 0.367 | 0.413 | 0.367 | 0.358 | 0.408 | 0.466 | 0.428 | 0.383 |
| 0.012 | 0.016 | 0.019 | 0.023 | 0.019 | 0.011 | 0.010 | 0.009 | 0.009 |
|  |  |  |  |  |  |  |  |  |
| 67.3 | 61.5 | 55.4 | 54.5 | 60.3 | 57.6 | 52.9 | 56.1 | 59.8 |
| 31.5 | 36.9 | 42.6 | 42.8 | 37.7 | 41.3 | 46.1 | 43.0 | 39.2 |
| 1.2 | 1.6 | 2.0 | 2.7 | 2.0 | 1.1 | 1.0 | 0.9 | 0.9 |

| **Plagioclase** | **Residual** |  |  |  |  |  |  |  |
| --- | --- | --- | --- | --- | --- | --- | --- | --- |
| **1080** | **1080** | **1080** | **1080** | **1080** | **1080** | **1080** | **1080** | **1080** |
| **2.0** | **2.0** | **2.0** | **2.0** | **2.0** | **2.0** | **2.0** | **2.0** | **2.0** |
| 52.91 | 52.56 | 52.24 | 53.19 | 53.02 | 54.34 | 51.87 | 50.44 | 50.73 |
| 0.19 | 0.12 | 0.14 | 0.02 | 0.2 | 0.15 | 0.09 | 0.08 | 0.02 |
| 28.52 | 28.03 | 28.14 | 28.47 | 26.21 | 27.17 | 28.78 | 28.86 | 29.4 |
| 1.78 | 1.76 | 1.86 | 1.17 | 2.39 | 1.55 | 1.5 | 2.22 | 1.59 |
| 0.05 | 0.02 | 0.06 |  | 0.07 | 0.06 |  | 0.02 |  |
| 0.23 | 0.24 | 0.29 | 0.12 | 1.75 | 0.25 | 0.24 | 0.31 | 0.21 |
| 12.15 | 12.47 | 12.63 | 11.66 | 11.35 | 10.73 | 12.42 | 13.42 | 13.25 |
| 4.36 | 4.22 | 4.06 | 4.67 | 4.27 | 4.95 | 4.18 | 3.72 | 3.86 |
| 0.13 | 0.18 | 0.14 | 0.15 | 0.16 | 0.22 | 0.14 | 0.1 | 0.1 |
| 100.32 | 99.60 | 99.56 | 99.45 | 99.42 | 99.42 | 99.22 | 99.17 | 99.16 |
|  |  |  |  |  |  |  |  |  |
|  |  |  |  |  |  |  |  |  |
| 2.409 | 2.413 | 2.401 | 2.431 | 2.441 | 2.481 | 2.387 | 2.340 | 2.344 |
| 0.007 | 0.004 | 0.005 | 0.001 | 0.007 | 0.006 | 0.003 | 0.003 | 0.001 |
| 1.530 | 1.517 | 1.525 | 1.534 | 1.422 | 1.463 | 1.561 | 1.578 | 1.601 |
| 0.068 | 0.068 | 0.072 | 0.045 | 0.092 | 0.059 | 0.058 | 0.086 | 0.061 |
| 0.002 | 0.001 | 0.002 |  | 0.003 | 0.002 |  | 0.001 |  |
| 0.016 | 0.016 | 0.020 | 0.008 | 0.120 | 0.017 | 0.016 | 0.021 | 0.014 |
| 0.593 | 0.613 | 0.622 | 0.571 | 0.560 | 0.525 | 0.612 | 0.667 | 0.656 |
| 0.385 | 0.376 | 0.362 | 0.414 | 0.381 | 0.438 | 0.373 | 0.335 | 0.346 |
| 0.008 | 0.011 | 0.008 | 0.009 | 0.009 | 0.013 | 0.008 | 0.006 | 0.006 |
|  |  |  |  |  |  |  |  |  |
| 60.1 | 61.3 | 62.7 | 57.4 | 58.9 | 53.8 | 61.6 | 66.2 | 65.1 |
| 39.0 | 37.6 | 36.5 | 41.6 | 40.1 | 44.9 | 37.6 | 33.2 | 34.3 |
| 0.8 | 1.1 | 0.8 | 0.9 | 0.9 | 1.3 | 0.8 | 0.6 | 0.6 |

| **Plagioclase** | **Residual** |  |  |  |  |  |  |  |
| --- | --- | --- | --- | --- | --- | --- | --- | --- |
| **1080** | **1080** | **1285** | **1285** | **1285** | **1285** | **1285** | **1285** | **1285** |
| **2.0** | **2.0** | **2.0** | **2.0** | **2.0** | **2.0** | **2.0** | **2.0** | **2.0** |
| 53.74 | 54.59 | 55.27 | 55.87 | 54.99 | 54.19 | 55.43 | 54.16 | 54.31 |
| 0.36 | 0.44 | 0.15 | 0.11 | 0.07 | 0.09 | 0.1 | 0.16 | 0.05 |
| 26.34 | 25.23 | 26.33 | 26.31 | 27.34 | 27.19 | 26.79 | 27.12 | 26.86 |
| 2.35 | 2.52 | 1.64 | 1.79 | 1.27 | 2.02 | 1.26 | 2.05 | 2.08 |
| 0.06 | 0.04 | 0.04 |  | 0.01 | 0.04 | 0.06 | 0.02 |  |
| 0.61 | 1.21 | 0.14 | 0.16 | 0.14 | 0.25 | 0.13 | 0.18 | 0.23 |
| 10.99 | 10.28 | 10.12 | 10.08 | 10.95 | 11.55 | 10.54 | 11.17 | 11 |
| 4.49 | 4.54 | 5.36 | 5.16 | 5 | 4.71 | 4.95 | 4.65 | 4.81 |
| 0.19 | 0.27 | 0.17 | 0.18 | 0.13 | 0.13 | 0.18 | 0.09 | 0.09 |
| 99.13 | 99.12 | 99.22 | 99.66 | 99.90 | 100.17 | 99.44 | 99.60 | 99.43 |
|  |  |  |  |  |  |  |  |  |
|  |  |  |  |  |  |  |  |  |
| 2.474 | 2.510 | 2.525 | 2.538 | 2.494 | 2.467 | 2.521 | 2.475 | 2.486 |
| 0.012 | 0.015 | 0.005 | 0.004 | 0.002 | 0.003 | 0.003 | 0.006 | 0.002 |
| 1.429 | 1.367 | 1.418 | 1.409 | 1.462 | 1.459 | 1.436 | 1.461 | 1.449 |
| 0.090 | 0.097 | 0.063 | 0.068 | 0.048 | 0.077 | 0.048 | 0.078 | 0.080 |
| 0.002 | 0.002 | 0.002 |  | 0.000 | 0.002 | 0.002 | 0.001 |  |
| 0.042 | 0.083 | 0.010 | 0.010 | 0.009 | 0.017 | 0.009 | 0.012 | 0.016 |
| 0.542 | 0.506 | 0.495 | 0.491 | 0.532 | 0.563 | 0.514 | 0.547 | 0.540 |
| 0.401 | 0.405 | 0.475 | 0.455 | 0.440 | 0.416 | 0.437 | 0.412 | 0.427 |
| 0.011 | 0.016 | 0.010 | 0.010 | 0.008 | 0.008 | 0.010 | 0.005 | 0.005 |
|  |  |  |  |  |  |  |  |  |
| 56.8 | 54.6 | 50.5 | 51.4 | 54.3 | 57.0 | 53.5 | 56.7 | 55.6 |
| 42.0 | 43.7 | 48.5 | 47.6 | 44.9 | 42.1 | 45.5 | 42.7 | 43.9 |
| 1.2 | 1.7 | 1.0 | 1.0 | 0.8 | 0.8 | 1.0 | 0.5 | 0.5 |

| **Plagioclase** | **Residual** |  |  |  |  |  |  |  |
| --- | --- | --- | --- | --- | --- | --- | --- | --- |
| **1285** | **1285** | **1285** | **1285** | **1285** | **1285** | **1285** | **1285** | **1285** |
| **2.0** | **2.0** | **2.0** | **2.0** | **2.0** | **2.0** | **2.0** | **2.0** | **2.0** |
| 55.22 | 55.48 | 55.99 | 56.61 | 57.37 | 55.17 | 54.96 | 54.4 | 54.08 |
| 0.13 | 0.11 | 0.07 | 0.04 | 0.1 | 0.06 | 0.08 | 0.08 | 0.11 |
| 26.87 | 26.84 | 27.09 | 25.5 | 25.85 | 27.05 | 27 | 27.31 | 27.07 |
| 1.87 | 1.81 | 1.22 | 1.74 | 1.53 | 1.35 | 1.48 | 1.79 | 2.09 |
|  | 0.01 |  | 0.03 |  | 0.03 |  | 0.05 | 0.01 |
| 0.19 | 0.15 | 0.15 | 0.11 | 0.13 | 0.11 | 0.15 | 0.12 | 0.35 |
| 10.64 | 10.67 | 10.45 | 9.39 | 10.37 | 10.61 | 10.53 | 11.17 | 11.39 |
| 5.09 | 5.01 | 5.03 | 5.4 | 4.97 | 5.03 | 5.08 | 4.72 | 4.64 |
| 0.1 | 0.13 | 0.23 | 0.16 | 0.15 | 0.16 | 0.13 | 0.13 | 0.1 |
| 100.11 | 100.21 | 100.23 | 98.98 | 100.47 | 99.57 | 99.41 | 99.77 | 99.84 |
|  |  |  |  |  |  |  |  |  |
|  |  |  |  |  |  |  |  |  |
| 2.504 | 2.512 | 2.524 | 2.582 | 2.577 | 2.509 | 2.505 | 2.479 | 2.469 |
| 0.004 | 0.004 | 0.002 | 0.001 | 0.003 | 0.002 | 0.003 | 0.003 | 0.004 |
| 1.436 | 1.432 | 1.440 | 1.371 | 1.369 | 1.450 | 1.450 | 1.467 | 1.457 |
| 0.071 | 0.069 | 0.046 | 0.066 | 0.057 | 0.051 | 0.056 | 0.068 | 0.080 |
|  | 0.000 |  | 0.001 |  | 0.001 |  | 0.002 | 0.000 |
| 0.013 | 0.010 | 0.010 | 0.007 | 0.009 | 0.007 | 0.010 | 0.008 | 0.024 |
| 0.517 | 0.518 | 0.505 | 0.459 | 0.499 | 0.517 | 0.514 | 0.545 | 0.557 |
| 0.448 | 0.440 | 0.440 | 0.478 | 0.433 | 0.444 | 0.449 | 0.417 | 0.411 |
| 0.006 | 0.008 | 0.013 | 0.009 | 0.009 | 0.009 | 0.008 | 0.008 | 0.006 |
|  |  |  |  |  |  |  |  |  |
| 53.2 | 53.6 | 52.7 | 48.5 | 53.0 | 53.3 | 52.9 | 56.2 | 57.2 |
| 46.1 | 45.5 | 45.9 | 50.5 | 46.0 | 45.8 | 46.2 | 43.0 | 42.2 |
| 0.6 | 0.8 | 1.4 | 1.0 | 1.0 | 0.9 | 0.8 | 0.8 | 0.6 |

| **Plagioclase** | **Residual** |
| --- | --- |
| **1285** | **1285** |
| **2.0** | **2.0** |
| 54.45 | 53.53 |
| 0.06 | 0.03 |
| 26.86 | 27.5 |
| 2.01 | 1.57 |
| 0.07 | 0.07 |
| 0.18 | 0.13 |
| 11.24 | 11.4 |
| 4.56 | 4.59 |
| 0.22 | 0.12 |
| 99.65 | 98.94 |
|  |  |
|  |  |
| 2.488 | 2.461 |
| 0.002 | 0.001 |
| 1.446 | 1.490 |
| 0.077 | 0.060 |
| 0.003 | 0.003 |
| 0.012 | 0.009 |
| 0.550 | 0.562 |
| 0.404 | 0.409 |
| 0.013 | 0.007 |
|  |  |
| 56.9 | 57.5 |
| 41.8 | 41.8 |
| 1.3 | 0.7 |
